# Validation, Calibration and Transparency, More than Accuracy: An Appraisal of Artificial Intelligence Against Antimicrobial Resistance and a Sustainable, Testable Diagnostic Architecture

**DOI:** 10.3390/microorganisms14102148

**Published:** 2026-09-23

**Authors:** Viktor Gulyás-Oldal, Imre Gálóczi, Ekaterine Paschali, Dániel Sallai, Csilla Dézsi, Péter Tamás Mészáros, Dezső Virok, Szabolcs Várbíró, Sándor Nagy, Károly Szili

**Affiliations:** 1 Department of Obstetrics and Gynecology, Faculty of Health and Sport Science, Széchenyi István University, Egyetem tér 1, 9026 Győr, Hungary; goviktor93@gmail.com (V.G.-O.); sallai.daniel@yahoo.com (D.S.); sziline.dezsi.csilla@szte.hu (C.D.); meszarosp.7@gmail.com (P.T.M.); 2 Doctoral School of Regional Sciences and Business Administration, Széchenyi István University, Egyetem tér 1, 9026 Győr, Hungary; 3 Department of Obstetrics and Gynecology, Faculty of Medicine, University of Szeged, Semmelweis utca 1, 6725 Szeged, Hungary; varbiro.szabolcs@med.u-szeged.hu; 4 S.O.S. 24 Kft., Kormányos utca 5, 6725 Szeged, Hungary; virok.dezso.peter@med.u-szeged.hu; 5 Department of Clinical Microbiology, Faculty of Medicine, University of Szeged, Semmelweis utca 1, 6725 Szeged, Hungary

**Keywords:** sustainability, antimicrobial resistance, artificial intelligence, machine learning, clinical prediction models, PROBAST, TRIPOD, antimicrobial stewardship, molecular diagnostics, qPCR

## Abstract

Artificial intelligence is widely proposed against antimicrobial resistance, but how much of that literature could support clinical deployment is unclear. We searched PubMed (2016–2026) across four domains, retrieving all 2446 records. Of 725 eligible studies, 80 were appraised at full text against the artificial-intelligence appraisal extensions (PROBAST+AI, TRIPOD+AI). Selection favoured externally validated work, so the validation figures are upper bounds. Output grew from 10 studies in 2016 to 142 in 2025; within the appraised set there was no evidence that genuinely external evaluation became more likely (odds ratio 1.08/year, 95% CI 0.89–1.34). Of the 80, 45% were externally validated, 24% released a model a reader could apply, 23% assessed calibration, 16% justified a sample size, and five compared performances across patient characteristics against eight across sites; 8.8% called a random split external validation. Risk of bias was low in 16, unclear in 55 and high in nine, and none of the 18 deep-learning studies reported calibration (95% CI 0–17.6%, a loose bound on 18). We derive the design constraints these impose on a culture-independent diagnostic platform, a projection with no performance data. Validation, calibration and transparency limit this field more than accuracy does: discrimination was reported in every study appraised.

## 1. Introduction

Antimicrobial resistance is one of the largest infectious causes of death measured in the most recent global burden analysis, which attributes 1.14 million deaths directly to bacterial antimicrobial resistance in 2021, associates 4.71 million deaths with it, and forecasts 1.91 million attributable deaths annually by 2050 [1]. Where it ranks against other causes depends on how causes of death are attributed and grouped, so the estimate rather than the ranking is quoted here. These estimates have themselves been challenged on methodological grounds, and the widely quoted projection of ten million annual deaths has been shown to rest on assumptions that do not survive scrutiny [2]; the direction of travel, however, is not in dispute.

That burden is not contained by the clinic, and it is not only a clinical problem. Resistance genes move between people, animals and the environment, so selection pressure applied in one compartment is paid for in another, and human medicine, agriculture and the environmental reservoir are now read as one connected system rather than three [3]. Resistance in food-producing animals has risen steeply across low- and middle-income countries since 2000, with the sharpest increases in the regions least equipped to detect them [4]. The international response has shifted accordingly, from exhortation to quantities that can be audited: ahead of the high-level 2024 United Nations General Assembly meeting, the problem was reframed around measurable targets for sustainable access to effective antibiotics, for appropriate use, and for the infections that make antibiotics necessary in the first place [5]. Diagnostics occupy a particular position in that framing. Most antibiotic prescribing is empirical, and empirical prescribing is what diagnostic delay compels; a test that shortens the interval to a specific answer is therefore an antimicrobial-sparing intervention as much as a diagnostic one, and its value is counted in exposure avoided as well as in organisms named. Whether artificial intelligence can make that shortening reliable enough to act on is the question this review asks.

The clinical mechanism that sustains resistance is well understood and stubbornly hard to interrupt. A patient presents with a suspected infection, the causative organism is unknown, and conventional microbiology commonly requires 24 to 72 h to identify it and to report susceptibility. That interval is not universal—it is shorter where a rapid molecular panel or direct-from-positive-blood-culture identification is available and longer for fastidious organisms, mycobacteria and fungi—but it is the interval most patients are managed across. During that interval, the clinician prescribes for coverage rather than for evidence, and broad-spectrum therapy is the rational individual choice even when it is the wrong collective one. Every hour of diagnostic uncertainty is therefore paid for in selection pressure. Guidelines encode this trade-off rather than resolving it [6], and stewardship programmes work by reducing the duration of unnecessary exposure rather than by preventing the initial empirical decision [7].

Artificial intelligence is frequently proposed as the instrument that will break this cycle, and the proposal is plausible in principle. Machine learning can infer a resistance phenotype from a genome sequence rather than growing an organism for a day [8,9]; it can estimate the probability that a given patient carries a resistant organism before any culture returns [10]; it can rank empirical regimens against a patient’s own risk profile [11]; and it can extract a species or a resistance signal from a mass spectrum or a Raman trace that a human reader could not interpret [12,13]. Each of these has been demonstrated, and the studies cited here are among the better-conducted examples in the corpus appraised below. What is far less clear is how many of these demonstrations would survive contact with a different hospital, a different population or a different year, and whether any of them has been carried far enough that a clinician could act on the output.

Four terms recur below and are used throughout in one sense each. Artificial intelligence is the widest: any computational method that performs a task normally requiring human judgment. Machine learning is the subset that learns its rule from data rather than having it specified, and deep learning the subset of machine learning that uses multi-layer neural networks; every deep-learning study in this review is therefore also a machine-learning study and an artificial-intelligence study, so the labels are not alternatives and the model-class counts reported in Section 3.2 overlap rather than partition the corpus. Prediction model and classifier describe what the fitted object does—estimate a probability, or assign a class—irrespective of which of those families produced it. Clinical decision support describes not a model but a deployment: a model placed where a clinician will see its output. A study can therefore be all of these at once, and the appraisal instruments used here apply to the prediction model inside it, not to the label its authors chose.

That question is answerable, because the instruments for answering it now exist. The Prediction model Risk Of Bias Assessment Tool extended for artificial intelligence (PROBAST+AI) provides a structured assessment of the quality of model development and the risk of bias in model evaluation, together with applicability to a stated clinical question [14]. The Transparent Reporting of a multivariable prediction model for Individual Prognosis Or Diagnosis, extended for artificial intelligence (TRIPOD+AI), specifies what a prediction-model study must report for a reader to be able to judge it [15]. Both were developed by the same working group and are explicitly intended to be used together: one asks whether the science is trustworthy, the other whether the paper said enough for that judgment to be possible.

This is not the first attempt to take stock of the field, and the six most directly comparable efforts are set beside this one in Table 1. Tang and colleagues pooled the discrimination of machine-learning models for antimicrobial resistance across 25 studies and reported a summary area under the curve of 0.82, with evaluation outside the development data scarce [16]. Liu and colleagues appraised 162 models from 108 studies of colonisation or infection with multidrug-resistant organisms and found risk of bias concentrated in the statistical analysis, fewer than a quarter externally validated and only seven of those by an independent team [17]. Li and colleagues appraised 100 models from 62 studies of multidrug-resistant infection in adult intensive care, rating 87.1% at high risk of bias with external validation in 17 [18]; Pu and colleagues, on the same clinical question, pooled an area under the curve of 0.867 across 37 models and reported that most lacked calibration and external validation [19]. Weis and colleagues reviewed machine learning applied to matrix-assisted laser desorption/ionisation time-of-flight spectra and found that 4 of 36 studies evaluated their algorithm on an external dataset [20], and Pennisi and colleagues pooled predictive performance across 80 stewardship studies under QUADAS-AI [21]. A recent narrative review maps what artificial intelligence might contribute across infectious diseases as a whole, against the targets set at the 2024 United Nations General Assembly meeting, without appraising the studies it describes [22].

Three of them appraised risk of bias with PROBAST and reached conclusions that converge with ours: bias driven by the statistical analysis, external validation in a minority, and rarely reported calibration. That they drew on different studies under different eligibility rules is part of why we read the agreement as a property of the field. Each, however, examines a single application niche or surveys the field without appraising it; to our knowledge none applies the artificial-intelligence extensions of these instruments, published in 2024 and 2025, and none asks whether a study’s own description of its validation design matches what the study did. All seven were retrieved by the search described in Section 2.2 and excluded at screening as non-primary reports (File S3), which is how a further one would have surfaced; no separate systematic search of the review literature was made, so this is the comparable work the search returned rather than necessarily all of it.

The gap this review addresses, and what it adds. The existing reviews answer how well these models discriminate within one application niche. None of them answers how much of this literature could be deployed, and that question is now answerable in a way it was not two years ago. Three things are new here. First, the four domains are viewed together. Genomic prediction, clinical risk models, therapy and dosing, and rapid diagnostics are reviewed separately and behave differently; read side by side under one rule they show that the shortfall is common to all four and is therefore a property of the field rather than of any one application. Second, the artificial-intelligence extensions are applied. PROBAST+AI and TRIPOD+AI were published in 2025 and 2024, and we are not aware of a previous review of this literature that applies them; they ask questions—about model availability, about calibration, and about who the model was fitted for—that their predecessors do not. Third, a reading of what the studies’ own words describe is included. Whether an author’s description of their validation design matches what they did is not something an abstract-level search can establish, and it turns out to be the single item on which the field is least reliable: 8.8% of the appraised studies apply the term external validation to a random split of one dataset.

This review has two objectives. The first is empirical: to map ten years of prediction-model research relevant to antimicrobial resistance and to appraise a stratified core of it against PROBAST+AI and TRIPOD+AI, so that the field’s real methodological state is described with numbers rather than impressions. The second is constructive: to use those findings as design constraints for a diagnostic and therapeutic platform, and to show what it would mean to build such a system so that it is sustainable—in antimicrobial, economic, environmental and geographic terms—rather than merely fast.

## 2. Materials and Methods

### 2.1. Design

This is not a registered systematic review, and it is not a systematic review with meta-analysis. It is a structured evidence map and methodological appraisal: a narrative review conducted under a systematic and reproducible search strategy, reported with a Preferred Reporting Items for Systematic Reviews and Meta-Analyses (PRISMA) 2020 flow diagram [23]. The distinction matters enough to be drawn part by part. Systematic: the search, the deduplication, the two-stage screening, the eligibility criteria, the pre-specified selection of the appraisal core set, and the appraisal itself against PROBAST+AI and TRIPOD+AI all follow a rule fixed in advance, and every decision is recorded per study. Narrative: the synthesis. No effect estimate is pooled, and none should be: the studies differ in organism, specimen, outcome definition and reference standard to a degree that makes a summary discrimination statistic a number without a referent, which is why the review reports proportions of methodological practice rather than pooled performance. Constructive rather than synthetic: Section 4 is neither. It is a design specification derived from the appraisal’s findings, and it is argued rather than evidenced; Section 4.1 states what that means for how it should be read.

A narrative synthesis was chosen deliberately over a full systematic review because the review has a second, constructive objective that a strict systematic-review format would not accommodate. The review was not registered, and no protocol was prepared. Three things therefore cannot legitimately be drawn from this design, and are not claimed anywhere in what follows: a pooled estimate of how well these models perform, because nothing is pooled; a prevalence estimate for the eligible literature, because the appraised 80 are a deliberately weighted stratum of the 725 and not a random sample of them (Section 2.5); and a causal reading of the temporal model, for the reason given in Section 3.1. What the design does support is a description of methodological practice in a defined and fully recorded stratum, with the direction of its bias stated. Supplementary File S17 answers all 27 PRISMA 2020 items and states where the review meets the systematic-review standard and where it does not. Six shortfalls are declared, and they are the six that matter most. Four are answered as not done—no protocol, no registration, no reporting-bias assessment and no certainty-of-evidence grading. The other two are no duplicate independent screening and no duplicate independent appraisal. These are recorded against items 8, 9 and 11 as declared departures rather than as unanswered items, because those items ask whether the process was described, and it is described in full. They are weaknesses of method rather than of reporting, and Section 5.5 treats them as such.

### 2.2. Search Strategy

PubMed was searched on 19 August 2026 for records published from 2016 onwards, using four domain-specific queries constructed around a common methodological vocabulary block (machine learning, deep learning, artificial intelligence, neural network, random forest, gradient boosting, support vector, prediction model, and classifier) combined with domain terms:

- D1, genomic resistance prediction: whole-genome sequencing, pangenome, k-mer, resistome, and genotype, combined with antimicrobial resistance, susceptibility or minimum inhibitory concentration.
- D2, clinical and electronic health record (EHR) risk models: electronic health record, clinical or risk prediction, bacteraemia, bloodstream, sepsis, urinary tract infection, and pneumonia, combined with multidrug-resistant, multidrug-resistant organism (MDRO), ESBL, MRSA or carbapenem terms.
- D3, artificial intelligence-guided therapy: the methodological block extended with clinical decision support and reinforcement learning, combined with antibiotic prescribing, antimicrobial stewardship, empirical therapy, dose optimisation, dosing regimen, therapeutic drug monitoring or pharmacokinetics.
- D4, rapid diagnostics: matrix-assisted laser desorption/ionisation time-of-flight (MALDI-TOF) mass spectrometry, Raman spectroscopy, Fourier-transform infrared (FTIR) spectroscopy, microscopy, melting curve, real-time or digital PCR, hyperspectral imaging, lateral flow, and biosensor, combined with resistance, susceptibility testing or pathogen identification.

Full query strings, hit counts and retrieval dates are given in Supplementary File S1. The search log was written at search time rather than reconstructed afterwards. All four queries were retrieved to exhaustion (2446 of 2446 matching records), so the identification stage was complete within PubMed. It was not complete across databases: Embase, Scopus, Web of Science and the grey literature were not searched, and no citation chasing was performed. This is the review’s largest coverage limitation and it is not evenly distributed. PubMed indexes the clinical and microbiological literature well, so the clinical risk-model and rapid-diagnostics domains are likely to be well covered; it indexes conference proceedings and the engineering and computer-science literature poorly, so work on genomic prediction and on algorithmic method that appears at machine-learning venues is systematically under-represented here. Section 5.5 states what follows for the conclusions.

### 2.3. Eligibility Criteria

Studies were eligible if they developed or evaluated a prediction, classification or decision model whose output was relevant to the management of antimicrobial resistance in humans. Eligible outputs included phenotypic susceptibility or minimum inhibitory concentration predicted from genotype; the probability that a patient carries or will develop infection with a resistant organism; identification of the causative organism or of a resistance determinant from a diagnostic signal; and recommendation of an antimicrobial agent or dose.

Studies were excluded if they were not primary reports (reviews, editorials, letters, comments, errata, conference abstracts, protocols, and preprints); if the modelled target was not an antimicrobial-resistance outcome (plasmid or virulence-factor classification, variant calling, horizontal gene transfer, and vaccine antigen prediction); if they concerned antimicrobial discovery or design rather than resistance management; if the framing was environmental, agri-food or veterinary; or if the resistance in question was viral. Eight studies that passed title and abstract screening were found at full text to fail these criteria. They were replaced in the appraisal core set by the next studies on the priority ranking, and—because every one of the eight was excluded on a ground that an abstract already carried—they were also removed from the abstract-level evidence map, which therefore holds 725 studies rather than 733. The reasons are recorded per study in Supplementary Table S2.

### 2.4. Screening

Records were deduplicated by PubMed identifier and screened in two stages. An automated stage excluded records by publication type and applied topic-relevance rules derived from a manual reading of the retrieved titles; a manual stage reviewed the resulting candidate pool. Because an initial rule set was found to exclude decision-support and large language model studies that met the eligibility criteria, all automatically excluded records were re-screened against a broadened methodological vocabulary, and 31 additional studies were recovered and included. All screening decisions, with reasons, are recorded in Supplementary File S3.

### 2.5. Selection of the Appraisal Core Set

Full-text appraisal of all included studies was not feasible. A core set of 80 studies, 20 per domain, was selected by a pre-specified priority score combining evidence of external validation (weight 3), evidence of prospective or implemented evaluation (weight 2), and citation rate per year on a logarithmic scale; a retrievable full text was a practical requirement, and no more than five studies per pathogen or clinical target were admitted per domain so that the appraised set would not collapse onto a single organism. Of the 733 records eligible on title and abstract, a full text was available in PubMed Central for 524 and not for 209; retrievability was operationalised as that deposit, and a record without one was not then pursued through the publisher or by interlibrary request, so the 209 is a count of what was not readily retrievable rather than of what could not be obtained at all. The requirement was that the text be readable, not that it carry an open licence; on this corpus the two coincided, because all 80 appraised studies carry an explicit open-access licence, quoted per study in File S24. Two consequences follow and are stated here rather than left to the reader. The appraised set is 80 of 725, or 11%, so every full-text figure in Section 3 describes that stratum and not the eligible literature as a whole. And because the priority score rewards external validation, prospective evaluation and citation, the stratum is deliberately weighted towards the better-conducted end of the field. The proportions for the items the score rewards—external validation above all, and prospective or implemented evaluation—are therefore upper bounds, and the direction of that bias is known even though its size is not. For the remaining items, the score does not select based on them directly, so the bias is presumed to run the same way, because they travel with methodological care, but that is an expectation rather than a consequence of the sampling rule, and its direction cannot be guaranteed. Twenty per domain was chosen for tractability at full-text depth rather than derived from a precision target, and the consequence should be read alongside every domain-level figure below: at *n* = 20, a proportion of 3 in 20 carries a 95% Wilson interval of roughly 5% to 36%, which is why domain-level contrasts are reported with intervals and an exact test rather than as established differences. This is the same sample-size justification the review finds absent in 83.8% of the corpus, and it is absent here too.

### 2.6. Appraisal

Each core-set study was classified as development only, development plus evaluation, or evaluation only, and appraised at full text with PROBAST+AI across the four domains—participants and data sources, predictors, outcome, and analysis—for the development pass, the evaluation pass, or both, together with an applicability judgment against the review question [14]. Judgments were recorded as low concern, high concern or unclear, with a written rationale for each study; the per-study record is given in Supplementary Table S5.

Alongside the domain judgments, ten methodological facts were extracted by reading the full text: study type, the validation design actually performed, whether the study’s own description of that design was accurate, and whether calibration, sample-size justification, missing-data handling, class-imbalance handling, overfitting control and any fairness or subgroup analysis were present.

Three of those items needed a rule stated in advance, because the answer depends on where the boundary is drawn rather than on what the paper says. Validation design follows TRIPOD+AI: an evaluation is external if the evaluation data differ from the development data in place (another centre, health system, region or country), in time (a later period held out and evaluated as such, or a prospective period after the model was locked), or in source (a separately assembled or separately published dataset). A random or stratified split of one assembled dataset is internal however it is named; cluster- or group-wise resampling of one assembled dataset—leave-one-site-out, leave-one-cluster-out, internal–external cross-validation—is internal for the same reason, because the model is refitted for every partition and no data outside the assembled corpus is involved [15]; and a model re-fitted on the new data is not being externally validated at all. This is the TRIPOD+AI reading, and it is not the only defensible one. A looser convention, common in the prediction-model literature, treats internal–external cross-validation—leave-one-site-out over a multi-centre dataset—as external, on the argument that each fold is evaluated on data from a centre it was not fitted on; the methodological statement usually cited for it presents internal–external cross-validation as a legitimate design in its own right rather than as a weaker substitute [24]. We do not, because the model is refitted for every partition and no data outside the assembled corpus is ever involved, so the procedure estimates how well the method transports rather than how well this model does; a reader who wants to apply the published model cannot obtain that number from it. The consequence of choosing the stricter reading is worth stating plainly: it moves studies out of the external category and therefore lowers the headline proportion. Three studies in the appraised set rest on cluster- or group-wise resampling and are counted internal on this rule; a convention that admitted leave-one-centre-out resampling as external would move at least two of them the other way and raise the 45% reported in Section 3.4. The third resamples over strains rather than centres, and uses the resampling to choose the model, so a looser convention would not obviously admit it either. The rule chosen here is the conservative one, its effect is in the direction of understating the field, and every affected study is identifiable in File S23. Supplementary File S23 records which clause, or clauses, decided each of the eighty calls, so the rule can be checked in both directions: three studies rest on cluster resampling and are counted internal, while a fourth, separate study, which uses leave-one-strain-out cross-validation, is counted external, because the model trained on one cohort is evaluated on two separately assembled others. Subgroup performance was counted as present when a performance metric was reported separately for two or more groups and compared; reporting baseline characteristics by group, using a group variable as a predictor, or breaking performance down by antibiotic, organism or model class does not count. Because a decomposition by study site is a subgroup analysis in one reading and simply the external-validation design in another, site-level comparisons are counted separately from comparisons across patient characteristics, and both figures are reported in Section 3.6. Sample-size justification counts only where the justification concerns the sample the model was developed or evaluated on, which is what the prediction-model framework this review measures the item against asks for [25]; a study that sizes some other analysis of its own—an implementation audit or a prevalence survey—has not answered it, and one study was recoded on that ground after the item was stated (appraisal/rule_recodes.csv). Six of these items admit a partial answer—a calibration plot without an intercept and slope, a sample size defended in words without a computation, a class imbalance named but not acted on—and partial was counted as present throughout, which makes the reported proportions upper bounds. How much of each proportion is partial is itself informative, so it is given rather than left in the record. Of the 60 studies counted as addressing overfitting, 26 did so fully and 34 partially; of the 30 counted as handling class imbalance, 10 fully and 20 partially; and of the 25 counted as handling missing data, 20 fully and 5 partially. On the three remaining items the split is narrower: of the 18 counted as assessing calibration, 17 fully and one partially; of the 13 counted as justifying a sample size, 5 fully and 8 partially; and of the 13 counted as comparing performance across subgroups, 8 fully and 5 partially. The two items whose totals rest most heavily on partial answers are therefore the two highest of them, overfitting and class imbalance, and neither should be read as the proportion of studies that handled the problem properly. Every item count in Section 3.6 keeps the denominator at 80, which is what makes the items comparable with one another; where a count elsewhere in this review rests on a smaller denominator, the denominator is printed with it. Supplementary Table S5 carries the per-study codes, and a blank cell there is an item the coder recorded as not applicable to that study: 34 studies for missing-data handling, 36 for class imbalance and 4 for overfitting control, and none on any other item. Holding the denominator at 80 therefore counts not applicable alongside not done on those three, which pushes their printed proportions down; among the studies to which the item does apply, missing data were handled in 25 of 46 (54.3%), class imbalance in 30 of 44 (68.2%) and overfitting in 60 of 76 (78.9%). Both readings are given in File S14, and Section 3.6 quotes the all-80 figures so that every item there shares one denominator.

TRIPOD+AI reporting items with unambiguous textual signatures—ethics and consent, funding, conflicts of interest, data availability, code availability, protocol, registration, patient and public involvement, and consideration of health inequalities—were coded by rule-based full-text search with negation and aspiration handling, so that a sentence such as “external validation is warranted” could not be counted as external validation having been performed [15]. This automated coding was validated against manual reading of a random sample of eight studies. The validation showed that the items determining the review’s central claims—validation design and calibration—could not be coded reliably by text search, because several papers use the term external validation for a random split of their own data. Those items were therefore human-coded for all 80 studies, and the automated coding was retained only for the mechanical reporting items.

Model availability was subsequently changed to human coding as well, for all 80 studies, after external methodological audit showed that text search failed on it in both directions. It missed studies that print their model in full, because the patterns required particular verb forms and matched neither “a nomogram model was constructed” nor a printed regression equation; and it counted two studies whose web tool was not their own, one of them a service used to draw a figure of the network. A pattern cannot tell whose artefact a sentence describes. The item is defined here as an artefact from which a reader can obtain a prediction for a new case without refitting anything—a printed equation with every coefficient, a nomogram, a complete points table, a runnable calculator, or a deposited fitted model—which is what distinguishes TRIPOD+AI item 18e from item 18b, code availability, reported separately in Section 3.6. Released analysis or model-training code does not qualify, however complete, because it lets a reader re-derive a model rather than compute a prediction. Supplementary File S21 gives the definition, the artefact class and the deciding quotation for every study, and the coding script fails the build if any study coded negative carries a candidate signal without a written reason.

Because a single coder is this review’s central methodological weakness, the items on which its conclusions rest were then coded a second time for all eighty studies: the validation design actually performed, the accuracy of the study’s own description of it, calibration, and subgroup performance with the patient-versus-site split. The second pass was made from full text under the rules stated above by a large language-model agent given the papers and the rules but not the original codes, and it was completed before any of the figures in Section 3 were written. Each of the 320 comparisons is recorded in Supplementary Table S16b with a quotation and a reason on each side; the 23 disagreements were adjudicated in writing against the full text, and the master record carries the ruling and the rationale for each. This is not a second human reviewer and is not offered as one. It is a documented, independently started machine re-coding with written adjudication, and what it establishes is which items are robust to who is reading—not how far a human duplicate would have agreed. The blinding was also only partial, and is reported as such: the coder had already seen the original code of 39 of the 80 studies, and Table S16b flags them so the two halves can be compared.

### 2.7. Analysis

Descriptive statistics were computed over the included set at abstract level (the evidence map, n = 725) and over the core set at full text (the appraisal, n = 80). Every proportion is reported with a Wilson score 95% confidence interval; Wilson rather than Wald because several of the quantities of interest lie close to zero, and because the per-domain denominators are 20, where a Wald interval leaves the unit interval. Comparisons between domains are Fisher exact tests with two-sided *p*-values, since the smallest cells are single digits. Analyses were performed in Python 3.12 with pandas 3.0 and SciPy 1.17. The two scripts that produce every proportion and every model estimate in Section 3 are supplied verbatim: File S20 writes the proportions and their intervals, File S20b the temporal model, so that each figure in Section 3 can be recomputed from the appraisal records rather than taken on trust.

Abstract-level figures are reported as such throughout. For most items they are a lower bound, because an item absent from an abstract may still be present in the paper, and the gap between the abstract-level and full-text estimates of the same quantity is itself reported, since it measures what authors consider worth putting in an abstract. The external-validation item is the one exception and errs in both directions at once: an abstract may omit a validation that was performed, and it may equally use the term for a procedure that was not one. It should therefore be read as a measure of external-validation language, and the full-text coding in Section 3.4 is what the review’s claims about validation rest on.

The temporal analysis was a logistic regression of the binary validation indicator on year of publication, with 95% intervals from the profile likelihood and *p*-values from the likelihood-ratio test. Two specifications were pre-specified: the primary one over complete years of publication only, and a sensitivity analysis over all years including the partial year 2026. The primary specification excludes 2026 because the search was run on 19 August of that year, so its records are a sample of eight months rather than of a year, and because a partial year is not comparable with the complete years around it. Both are reported in Section 3.1; they disagree, and the reason they disagree is reported with them.

## 3. Results

### 3.1. Study Selection and Growth of the Field

The search identified 2446 records; 2193 remained after removal of duplicates; 1460 were excluded at screening; and 733 met the eligibility criteria on title and abstract. Eight of those were found at full text to fail the criteria of Section 2.3—two imported-malaria papers, a veterinary surveillance study, a honey-bee disease study, a sewage-metagenome analysis, a conference-abstract supplement, a study protocol and a docking analysis. Every one of those grounds is decidable from an abstract, so they are screening misses rather than findings that only the full text could produce, and they are removed from the evidence map as well as from the appraisal: 725 studies carry the abstract-level analysis (Figure 1). The record counts behind the diagram are given in Supplementary File S1b and the eight exclusions, with reasons, in Supplementary Table S2.

The largest excluded category was records that did not describe an artificial intelligence or machine learning prediction model on an antimicrobial-resistance-relevant target (n = 879), followed by non-primary reports (n = 490); the remaining exclusions are itemised in Figure 1.

Included studies were unevenly distributed across the four domains: clinical and electronic health record risk models 275 (37.9%), genomic resistance prediction 176 (24.3%), rapid diagnostics 147 (20.3%) and artificial intelligence-guided therapy 127 (17.5%). Output grew steeply, from 10 eligible studies in 2016 to 142 in 2025, with 174 already indexed in the first eight months of 2026 (Figure 2). Growth was monotonic from 2017 onwards; the single fall in the series is the first one, from 10 in 2016 to four in 2017, on counts small enough that little should be read into it.

Growth was fastest in the clinical domain, which produced more eligible studies in 2025 alone than in the six years to 2021 combined.

Whether the evidentiary standard rose alongside that volume is the review’s one inferential question, and answering it honestly requires saying which of two disagreeing specifications we believe and why (Figure 2b). A logistic regression of external-validation mention on year of publication, fitted over the 551 studies published in complete years, gives an odds ratio of 1.04 per year (95% CI 0.91–1.21, *p* = 0.56). Fitted over all 725, including the partial year 2026, the same model gives 1.23 per year (1.10–1.39, *p* = 0.0001)—a significant increase. The two are irreconcilable, and the whole of the difference sits in the eight months of 2026, where the abstract-level mention rate is 23.6% against 8.3% across the preceding decade.

That step is not explained by what the corpus is made of. It is present within all four domains, it is present within the 56 journals that appear in both periods (7.6% rising to 28.7%), and the abstracts are only 7% longer. What it most plausibly reflects is the arrival of the reporting guidelines themselves: TRIPOD+AI was published in 2024 and PROBAST+AI in 2025, and a checklist vocabulary is adopted faster than a study design is changed. The abstract-level indicator, in other words, measures external-validation language, and that language changed.

The temporal claim of this review therefore rests on the one series in which validation design was read from the full text rather than matched in an abstract. Across the 70 appraised studies published in complete years—the partial year is set aside here on the same rule as above—the odds of a genuinely external evaluation changed by 1.08 per year (95% CI 0.89–1.34, *p* = 0.44): no evidence of an increase, in an interval still wide enough to accommodate one of about a third per year. Adding the ten studies from 2026 changes the estimate to 1.11 (0.93–1.34, *p* = 0.24) and changes nothing that matters. Growth in this field has been in volume and, more recently, in vocabulary; the practice those words describe has not been shown to have changed. All four fits are in Supplementary Table S15, and the diagnostics behind them—the domain, journal and abstract-length decompositions of the 2026 step—are printed by the script that produces it, supplied as File S20b.

One qualification belongs with that estimate rather than in the limitations, because it bears on what the estimate can mean. The 80 studies were chosen by a priority score that gave its heaviest weight to evidence of external validation (Section 2.5), so the outcome being regressed on year is the same quantity that governed entry to the sample. Conditioning on an outcome distorts an association with it in a direction that cannot be signed without knowing how the weight interacted with year, and the appraised series is in any case not a random sample of the field. What this fit supports is therefore narrower than a field-level trend: within a set of studies selected for being among the better-validated of their years, the proportion genuinely validated externally did not rise. Read as a check on the abstract-level series it is informative, because the selection rule did not change over the decade; read as an estimate of how the field changed it would be overreaching, and we do not offer it as one.

Publication was highly dispersed across 274 journals: the largest single contributor, Scientific Reports, accounted for 33 studies (4.6%), and no journal accounted for more than 5% of the corpus. This dispersion matters for interpretation, because it means the field has no small set of venues in which methodological standards could be raised by editorial action alone.

### 3.2. Methods and Validation, at Abstract Level

Regularised or classical regression appeared in 31.2% of included studies, tree ensembles in 30.1%, deep learning in 20.3% and support vector machines in 8.3%. Large language models appeared in 1.5%, entirely since 2024, and reinforcement learning in a single study. The distribution is notable for what it does not show: despite the prominence of deep learning in the field’s rhetoric, the modal model in this literature is a regression or a gradient-boosted tree on tabular data. Table 2 sets the classes side by side, at abstract level across all 725 studies and at full text across the 80 appraised.

Table 2 sets out a gradient, and the full-text column shows it to be narrower than the abstract-level column makes it look. At abstract level, every item appears to fall away as the model class becomes more flexible: regularised regression mentions external validation in 18.6% of abstracts and calibration in 40.7%, deep learning in 7.5% and 2.0%. Read at full text, external validation is close to flat—48.0% for regression, 48.4% for tree ensembles, 44.4% for deep learning and 60.0% for support vector machines—so the apparent gradient in that item is a gradient in what abstracts say rather than in what studies did. Calibration is the item that really is graded, and steeply: 52.0% of the appraised regression studies assessed it, 22.6% of tree ensembles, and 20.0% of support vector machines, and among the eighteen deep-learning studies appraised at full text, not one reported calibration of any kind (0 of 18; 95% CI 0–17.6%). The eleven large language model studies—all published since 2024—sit outside that comparison: none of them entered the appraised set, so what can be said of them is only that no abstract among the eleven mentions calibration. No ranking of the classes is offered on external validation, where the differences are well inside the intervals that twenty-five to thirty-one studies per class can support.

Two readings of this gradient are possible and they have different implications. It may be that flexible models are applied to problems where calibration is genuinely harder to define, such as multi-class species assignment from a spectrum, in which case the field needs guidance on what calibration means for those outputs. Or it may be that the culture around deep learning imported evaluation conventions from computer vision, where discrimination on a held-out split is the accepted currency and calibration is not routinely reported. The two are separable in principle: the first predicts that deep-learning studies with a binary outcome should report calibration at rates comparable to regression. In this corpus they do not, but the subgroup is too small to settle the question, and we report it as a direction rather than as a result. Two cautions belong with the gradient in any case. Model class is confounded with application domain and with data type—deep learning is overwhelmingly spectra and images in rapid diagnostics and regression is overwhelmingly tabular data in clinical prediction—and with 80 studies the two cannot be separated. Either way, a clinician reading a deep-learning resistance classifier is at present very unlikely to be told whether its probabilities mean anything.

At abstract level, 87 studies (12.0%, 95% CI 9.8–14.6) mentioned external validation, 94 (13.0%, 10.7–15.6) mentioned internal validation only, and 544 (75.0%, 71.8–78.0) mentioned neither. Calibration was mentioned in 16.0% (13.5–18.8), explainability methods in 15.6% (13.1–18.4), code availability in 2.8% (1.8–4.2), and any fairness or subgroup-equity analysis in 0.7% (0.3–1.6). For most items, these are lower bounds, and the comparison with the full-text figures in Section 3.4 quantifies how much lower. The external-validation item is the exception and runs in both directions at once: an abstract may omit a validation the paper performed, but it may equally use the term for a procedure that was not one, which is the subject of Section 3.4. The per-study extraction behind every abstract-level figure in this section is given in Supplementary Table S4, and every proportion with its interval in Supplementary Table S14.

### 3.3. The Appraised Core Set

The 80 appraised studies [8,9,10,11,12,13,26,27,28,29,30,31,32,33,34,35,36,37,38,39,40,41,42,43,44,45,46,47,48,49,50,51,52,53,54,55,56,57,58,59,60,61,62,63,64,65,66,67,68,69,70,71,72,73,74,75,76,77,78,79,80,81,82,83,84,85,86,87,88,89,90,91,92,93,94,95,96,97,98,99] were published between 2016 and 2026 across 57 journals, with a median of 13.5 citations. Sixty-five were development-plus-evaluation studies, 12 were development only, and three were evaluations of previously developed models. *Klebsiella pneumoniae* was the single most common target (16 studies), followed by sepsis and bloodstream infection (10), *Escherichia coli* (seven), urinary tract infection (six) and *Staphylococcus aureus* (six).

### 3.4. What Validation Was Actually Performed

Thirty-six studies (45.0%, 95% CI 34.6–55.9) evaluated their model on data external to the development set under the rule stated in Section 2.6, 43 (53.8%, 42.9–64.3) reported internal validation only, and one (1.3%, 0.2–6.8) reported apparent performance on the development data alone. That figure includes temporal and prospective evaluations at the developing institution as well as evaluations in another centre or another country, because TRIPOD+AI counts both; the temporal form is the weaker one, since it tests whether a model survives drift rather than whether it survives a different population. External validation was least common in the rapid-diagnostics domain (5 of 20; 25.0%, 11.2–46.9) and most common in the therapy domain (11 of 20; 55.0%, 34.2–74.2), but with twenty studies per domain that difference is not one the data can support (Fisher exact *p* = 0.105, odds ratio 3.67), and it is reported as an ordering rather than as a difference.

The most consequential finding of the appraisal concerns terminology. Seven studies (8.8%, 4.3–17.0) used the words external validation or independent for a procedure that was a random split of a single dataset. A further one claimed to have validated an approach across four species when the evaluation was an 80/20 split of one laboratory’s data, so that eight studies in all (10.0%, 5.2–18.5) gave their design a name it did not earn. One paper states that data “was partitioned into a training set (n = 744, 70%) and a hold-out test set (n = 319, 30%) to ensure robust external validation”; another reports 2683 isolates from a single hospital divided 80/20, with “the remaining 20% of data used as the independent data set for external validation”.

That 10.0% is the item on which the two independent codings agreed least, and it is reported here as the range the record supports rather than as a point. Counting every study whose own wording was judged inaccurate, the first coding gives 11 of 80 (13.8%), the independent second coding nine of 80 (11.2%), and the adjudicated record eight of 80 (10.0%); Cohen’s kappa for the item is 0.54 (Section 3.5). The adjudicated figure is the lowest of the three, which is what the account of that boundary in Section 5.5 would predict—where the two readings differed, adjudication against the full text more often upheld the study than the objection—so the number quoted above is the bound most favourable to the corpus. Even on that reading, one appraised study in ten gave its validation design a name it did not earn; on the least favourable reading in the record, closer to one in seven. All three codings, with the quotation behind each, are in Supplementary Table S16b.

This is not a semantic quibble. A reader—or an automated evidence synthesis—that accepts the word at face value will conclude that transportability has been demonstrated when nothing outside the development population has been examined. It also means that the abstract-level figure of 12.0% for external validation is not merely an underestimate of a true rate but a measurement of a term whose usage is unreliable.

Where transportability was genuinely tested, the results were sobering and, in the better papers, reported honestly. A unitig-based pangenome model for *Pseudomonas aeruginosa* fell from an internal area under the receiver operating characteristic curve (AUC) above 0.93 to approximately 0.77 on an independent published cohort [37]. A model developed on English *E. coli* genomes and applied to isolates from Uganda, Nigeria and Tanzania showed the performance loss its authors attributed to overfitting on the development data [8]. A study of MALDI-TOF prediction for *P. aeruginosa* reported the uncomfortable observation, rather than omitting it, that adding training data from external institutions degraded internal performance [88].

### 3.5. Risk of Bias and Applicability

How the appraisal was performed determines how these counts should be read. PROBAST+AI was applied at the level of its four domains—participants, predictors, outcome and analysis for development; and the corresponding evaluation domains—with a written rationale recorded for every study rather than as a published answer to each of the 16 development and 18 evaluation signalling questions; the underlying facts are given per study in Table S5 and the rationales in the same file. A single assessor made every judgment, which is the review’s principal methodological weakness (Section 5.5), so the reliability of the four judgments that carry the conclusions was measured rather than asserted: all 80 studies were re-coded from full text on validation design, the accuracy of the authors’ own description of it, calibration and subgroup performance, giving 320 comparisons of which 297 agreed (92.8%), with Cohen’s kappa of 0.90, 0.54, 0.74 and 0.86, respectively. Every one of the 23 disagreements was adjudicated in writing against the full text (File S16b). The two boundaries that proved hardest to hold—unclear against high, and an accurate description of a validation design against an inaccurate one—are exactly the two on which the distribution below should be read as conservative.

Overall quality of model development was rated as a low concern in 27 studies, unclear in 42 and high in eight. These three counts sum to 77, not 80, because the development part of PROBAST+AI does not apply to the three studies that evaluated a model developed elsewhere. The evaluation part, by contrast, was applied to all 80: a study described here as development only still evaluates its model on its own data, and that evaluation carries a risk of bias whether or not it reaches outside the development population. Overall risk of bias in model evaluation was accordingly low in 16, unclear in 55 and high in nine across all 80. Figure 3 shows both passes, domain by domain. Applicability to the review question is judged separately from either and is not in that figure: it was a low concern in 49 studies, unclear in 22 and high in nine, and the per-study judgment is in Table S5.

The domain-level pattern is consistent across all four fields (Figure 3). Domain 1 (participants and data sources) and domain 2 (predictors) were generally sound: clinical isolates and patient cohorts were usually appropriate, and predictors were usually derived uniformly. Domain 3 (outcome) was frequently unclear, most often because the phenotypic reference standard and the breakpoint version were not stated—a serious omission in a field where the label is defined by a versioned standard that changes [100]. Domain 4 (analysis) carried the great majority of the concern in both passes: low concern in 34% of studies on the development pass and 24% on the evaluation pass.

High-concern ratings clustered in studies whose sample was too small to support the claim being made. A neural network was trained on 685 protein sequences to classify resistance in *Porphyromonas gingivalis*, with labels derived from sequence annotation rather than any phenotypic test, and with the authors stating that dropout tuning and cross-validation were not performed [40]. A surface-enhanced Raman classifier for methicillin-resistant *S. aureus* was built from 19 MRSA isolates and a single susceptible isolate, with roughly 1500 spectra per isolate creating an apparent sample size that belongs to spectra rather than to organisms [13]. A model predicting the appropriateness of antibiotic treatment was developed on 105 patients containing 22 events, and then used to support mortality-stratification claims [47].

### 3.6. Reporting Completeness

Full-text reporting was better than abstracts suggested but remained poor in the areas that determine whether a model can be trusted or reused (Figure 4). A discrimination or accuracy metric was reported in every study, which is the one item on which the corpus is unanimous and the only one that needs no interval. Overfitting was addressed in 75% (64.5–83.2). Beyond that, the figures decline sharply: a data availability statement in 52.5%, class-imbalance handling in 37.5%, code in 33.8%, missing-data handling in 31.2%, the model itself in a form a reader could apply in 23.8%, calibration in 22.5%, a sample-size justification and any comparison of performance across subgroups in 16.2% each, registration in 7.5%, consideration of health inequalities in 3.8%, a protocol in 1.2%, and patient or public involvement in none. Model availability and code availability are counted separately and the gap between them is the point: 33.8% released analysis or training code, but only 23.8% released something from which a reader could obtain a prediction without refitting the model themselves (File S21).

Two of these deserve emphasis. Calibration, assessed in fewer than a quarter of studies, is not an optional refinement: a model whose predicted probabilities are miscalibrated may discriminate perfectly and still be unusable for any decision that depends on a threshold, which describes every decision in this field. The absence of sample-size justification in 83.8% of studies is equally consequential, since the standard framework for prediction-model sample size has been available throughout the review period [25] and was applied in only a handful of the studies appraised.

Fairness needs its definition stated before its number can be read, because the two plausible definitions differ by a factor of two. Thirteen studies (16.2%, 95% CI 9.7–25.9) reported a performance metric separately for two or more groups and compared them. In five of those (6.2%, 2.7–13.8) the groups were patient characteristics—age, sex, ethnicity, renal function, income setting—and the comparison was explicit, in one case with bootstrapped *p*-values for a difference in discrimination between age bands and between men and women [96]. In the remaining eight, the decomposition was by study site, laboratory or clinical syndrome, which is informative about transportability but is not an equity analysis and in several cases is simply the external-validation design reported per centre. On the stricter reading, then, fewer than one study in twelve asked whether its model works equally well for different patients, in a field whose burden falls disproportionately on populations that are least represented in its training data—and on either reading, nobody examined performance by socioeconomic position or by resource level of the treating institution.

### 3.7. Studies That Show What Good Practice Looks Like

The appraisal also identified a minority of studies that demonstrate the standard is achievable. A model predicting bacteriuria in emergency department patients was developed on eight years of data, compared three imputation strategies, was explicitly re-calibrated, and was validated on a temporally separate period, reaching an AUC of 0.813 (95% CI 0.792–0.834) [96]. A resistance-prediction model for Enterobacterales bloodstream infection was tested on a held-out later period, benchmarked directly against clinician prescribing, evaluated under a constrained-antibiotic-use scenario, and released with its analysis code [11]. A MALDI-TOF resistance-prediction study monitored performance prospectively for 18 months and reported how it decayed as the training data aged—to our knowledge, the only study in the corpus to measure model drift prospectively [12]. A multicentre Italian study quantified how much cross-centre generalisability was lost, treating site heterogeneity as a measurable quantity rather than a limitation paragraph [81]. A previously derived model for diarrhoea aetiology was validated prospectively in Bangladesh and Mali with a pre-stated sample-size calculation and both calibration-in-the-large and calibration slope reported [61]. A model for carbapenem-resistant organisms in postoperative intensive care assessed its sample size explicitly against the Riley framework [69].

These studies are not concentrated in any one domain, journal or country, which suggests the constraint is a methodological convention rather than a resource constraint.

## 4. A Proposed Sustainable Approach: The Live-Sample Platform, as a Design Specification

### 4.1. Design Rationale

The appraisal in Section 3 identifies a pattern rather than isolated defects: models in this field are numerous, discriminate well on the data that produced them, and are almost never carried to the point where a clinician could act on them. The bottleneck is not predictive accuracy but the absence of a deployable path from specimen to decision.

The platform described here is a design response to that gap, and deliberately not a new classifier. It is a workflow in which several small, auditable models sit where a decision must be made anyway, inside a laboratory process short enough to change the first antibiotic a patient receives rather than the third. Two commitments constrain everything below: every module must be specified against TRIPOD+AI before it is built and evaluated under an external, prospective design rather than the random split that 8.8% of the appraised corpus called external validation [14,15]; and the platform must be justifiable on resource grounds and not on accuracy alone, because a diagnostic that improves therapy while increasing reagent consumption, cost and waste has changed the problem rather than solved it.

Nothing in this section is a result. It is a proposed design derived from the appraisal—a design specification and a set of testable hypotheses, not a characterised device—carrying no analytical or clinical performance data of any kind. Every figure quoted below is derived in a Supplementary File so that it can be checked rather than believed, but derivable is not the same as demonstrated: a cost that follows correctly from a unit price is still a cost for an instrument that does not exist. The section is written so that each commitment can be falsified by a specific experiment, and Section 4.8 names the experiments.

The endpoints, in the order they must be satisfied. The review’s own finding is that this field mistakes an intermediate endpoint for a clinical one, so the platform states its hierarchy in advance. Analytical validity comes first and is not negotiable: limit of detection, inclusivity and exclusivity, inhibition, multiplex interference and carry-over, measured per target and per specimen matrix (File S22). Clinical validity comes second: prospective, multi-site agreement against a reference standard, with calibration as a primary endpoint rather than a supplementary plot, because every decision the platform makes is threshold-driven. Clinical utility comes third and is the endpoint that matters: whether the time to appropriate therapy falls, whether unnecessary broad-spectrum exposure falls, and whether patient outcomes are at least unchanged. Discrimination is an intermediate quantity throughout. No AUC is a primary endpoint anywhere in this design, and a module that reached an excellent one without clearing the first two stages would not be eligible for use.

### 4.2. Overall Architecture

The proposed platform would couple a culture-independent molecular workflow to seven model decisions, M0 to M6: determine the kingdom before any organism-level claim is released (M0, Section 4.3), choose which panel and which resistance targets are interrogated (M1 and M2), recommend an agent and a dose (M3 and M4), flag the patients in whom microbiota restoration should be considered (M5), and follow the patient afterwards (M6). The last is what this literature almost entirely omits—the appraised models overwhelmingly stop at the moment of prediction. Figure 5 sets out the architecture that follows.

The wet-laboratory core would be a lysis and releaser fluid applied directly to the primary specimen, followed by amplification on a six-channel real-time PCR instrument. The releaser is intended both to lyse bacterial cells and to deplete the human nucleic acid background, so that no purification step is required—which is what would compress the analytical phase to roughly an hour, and is the assumption most needing demonstration (Section 4.8). Assays are supplied lyophilised, the only liquid added at the bench being the releaser-treated specimen. The specification is not tied to any manufacturer; parameters and their provenance are in Supplementary Table S9, and the seven modules—the decision each replaces, the model class proposed, what each must show before use and the decision it may not make—module by module in Table S10.

Four choices follow from Section 3. Model class is chosen against the evidence: the more flexible the class, the less of what is needed to trust it gets reported, so M1, M2, M5 and M6 are regularised regression or gradient-boosted trees, M0 a threshold rule on cycle-threshold values and M4 a population pharmacokinetic model. M3 is not a language model: eleven studies in the corpus applied large language models, most often to therapy and dosing, and not one reported calibration. Calibration is a release criterion, not a reported statistic. Every module may decline, and each has one decision it does not make—the points at which a model error would otherwise become an act.

### 4.3. Kingdom Determination as the First Thing Measured

Every question the platform asks downstream presupposes a prior one: which kingdom is causing this illness? Practice answers it retrospectively, if at all, by the pattern of what grew; the platform is specified to answer it first, in the same run as everything else. The first position of every configuration run as a first pass is a group-determination reaction—broad-range 16S for bacteria, ITS2 via ITS86F/ITS4 for fungi, 18S under mammalian blocking for protozoa, an internal amplification control, a host cellularity control and a route-specific reserved channel. Viruses would deliberately not be a fourth channel, because they share no universal gene, and would enter through the reserved channel and an escalation path. The reaction would be load-bearing rather than informative: outside the one-hour sepsis pathway it is the highest-leverage antimicrobial-sparing step in the workflow; it makes a negative species panel interpretable, since a positive bacterial channel with negative species positions means an organism outside the tested menu rather than no organism; and it is what the selection models are ultimately answerable to, since their choice is only meaningful if the kingdom is what they assumed. It does not, however, gate that choice. The positions are committed at manufacture, before any result exists, so the kingdom reaction verifies the selection inside the same run rather than authorising it—the consequence the design owns, and Section 4.5 sets out what follows from it.

The evidence is cited claim by claim rather than as a block, because the claims are not equally supported. Broad-range 16S primer sets and their coverage limits are established [101,102], and the copy-number variability that makes a 16S signal a poor quantity estimator is catalogued genome by genome [103]. ITS2 amplification with ITS86F/ITS4 identifies fungi [104,105]—but that primer pair was developed for soil and environmental metabarcoding, not for real-time PCR on a clinical specimen; its transfer here is an assumption, not an inheritance. The protozoal channel is on the same footing, adapted from 18S eukaryotic markers and consensus-primer precedents because no validated pan-protozoan primer exists [106,107,108]. Reagent-borne background in low-biomass specimens is why the no-template control is a release criterion [109]. The clinical case for interrogating culture-negative specimens and for a viral escalation that removes an antibacterial indication rests on separate evidence again [110,111,112], as do the virulence menus [113,114,115,116]. What none of this validates is the combination. Each source supports one component, mostly in a non-clinical matrix; there is no published evaluation of these six channels multiplexed in a single reaction on a clinical specimen. The group-determination reaction is therefore a hypothesis stated here for testing, not a validated assay; the analytical work it requires is specified in File S22 and the channel assignments in File S7.

### 4.4. Panel Architecture

Because the reagents are lyophilised in advance, a strip would be manufactured as a configuration: a fixed set of occupied positions in an eight-position housing, each carrying its own positive and no-template control pair, each position a six-plex reaction. The models choose which configuration is taken from the shelf; they do not choose which positions are wetted, because the reagent is committed at manufacture. Four configurations and their site-specific variants can be selected as a first run—kingdom only where the question is which kingdom and nothing more, triage for stable presentations on the respiratory route where the result precedes the prescription, standard for hospitalised patients without a sepsis trigger, and critical for sepsis—and two further ones exist only as a reflex second run. All six are set out with their contents, occupied positions and intended use in Supplementary Table S9b, which is generated from the same configuration object that prices them in Section 4.6. Table S9 states the provenance of every design value behind both.

Three safeguards answer the safety problem adaptive selection creates. An always-on core: Every configuration carrying organism positions reserves its first for the targets whose omission would be immediately dangerous—*Staphylococcus aureus* with *mecA/mecC*, Enterobacterales with carbapenemase determinants, and on the respiratory route, *Legionella pneumophila*. The reservation is physical rather than a rule in software, so the models cannot remove a core target even if they fail. The kingdom-only and triage configurations carry no organism position and therefore no core block, which is why either may be selected only where the result precedes the prescription. A one-way escalation rule: Models may broaden but may not narrow below the syndrome-specific default when severity markers are present, so failure degrades towards the standard of care rather than below it. A declared indeterminate state: When the run returns no target and the patient meets sepsis criteria [117], the platform reports “no organism identified within the tested panel” together with the targets that were not tested, because a negative adaptive panel is not a negative result and must not read as one.

### 4.5. Time Budget and the Release Rule

On the robot-assisted route, the critical path totals 99 min and on the fully manual route, 142: 24 min of pre-analytical handling, 15 of reaction preparation, the 45 min run and 15 of post-analytical work, against 40, 30, 45 and 27. The step-by-step budget is in Supplementary File S6 and every combination of crossing patterns in File S6c.

A crossing at cycle 22 releases nothing. A target crossing threshold early is a reflex trigger: it starts the advisory chain and authorises a second configuration to be charged, but it is not a diagnostic release, and the run is not stopped. Two controls decide the matter and neither has finished at cycle 22. The no-template control is clean only so far; contamination frequently amplifies late, and a detectable no-template signal at any cycle invalidates the run, so a clean trace at cycle 22 is provisional rather than a result. The internal amplification control is deliberately set to cross near cycle 28 so that it does not compete with a low-copy target, so every channel still flat is uninterpretable. Nothing is reportable until the run has finished with all controls valid—a negative never was releasable early, and on this policy, a positive is not either. Stopping the first run would in any case give up the channels that had not yet crossed, and in a polymicrobial or undifferentiated specimen, those may carry a second pathogen: a diagnostic loss no gain in minutes justifies.

What the early crossing buys is the recharge: the second configuration is charged inside the 23 cycles the first run still has to perform, so a reflex second run adds 45 min rather than 50. Every path releasing on one configuration takes 99 min; a sequential resistance run takes 144. Two conclusions follow. Kingdom and species positions must sit in the same configuration, since carrying them together releases at 99, whereas a sequential run releases at 144; the design owns the consequence—positions committed before the kingdom result exists mean the kingdom call cannot gate the species selection—so M1 predicts the kingdom and plausible organism set from clinical data, and the kingdom position verifies that prediction inside the same run. The speed claim is about an assay run, not about a patient: at the one to ten colony-forming units per millilitre typical of bloodstream infection (Section 4.8) a crossing near cycle 22 is not the expected behaviour, and a target-negative completed run may be an analytical false negative rather than an absence, so the studies of File S22 must precede any use of a negative result to narrow therapy. For context, and the context does not flatter this proposal: multiplex respiratory panels span roughly an hour for the fastest cartridge systems to four or five for the slowest, so the quickest of them return a result sooner than the 99 min specified here, while blood-culture identification panels take around a day and are generally conditional on prior culture positivity [118,119,120]. Separately, and in different laboratories, automation has been reported to cut blood-culture turnaround from 97 to 53.5 h [121,122,123] and, in an economic evaluation of a different installation, to lower total laboratory cost by roughly 12% [124,125,126]; the two are not one result and should not be read as one. What this design is arguing is therefore not that it is the fastest, but that it is culture-independent, priced in single digits per specimen, and does not require the prior positivity that the blood-culture panels do.

None of this meets a clinical need in sepsis if read as a gate on the first dose, and it is not permitted to be one: guidance requires therapy within the hour in septic shock [6]. In sepsis, the empirical first dose precedes the result, and the platform does not delay it. What it changes is the second decision: 19% of patients with bloodstream infection receive empirical therapy discordant with the eventual susceptibility, with an adjusted odds ratio for in-hospital mortality of 1.46 (95% CI 1.28–1.66) and resistance the strongest predictor of discordance at 9.09 (7.68–10.76) [127,128,129]. The relevant quantity is time to the first appropriate dose, which culture leaves at 48 to 96 h and only if the organism grows. The sequence can be operated in Files S13 and S11, and Figure 6 traces the decision flow, including the point at which a second run becomes unavoidable.

### 4.6. Consumable Cost

The unit costs are the developers’ own reagent costs, not a price charged to a health system: instrument amortisation, labour, quality control, overhead and margin are excluded. Two drive everything—the releaser at 1.00 USD per specimen and a lyophilised position at 1.20 USD—and each configuration is priced by the positions it occupies. One point of accounting must be explicit: the controls are lyophilised into each configuration, so a specimen carries its own control pair, charged in full. That pair validates that strip and that lyophilisation lot, which is what allows a configuration to be run where there is no separate quality-control infrastructure, and it is why the number of runs matters as much as the number of targets. Shared across a run of 48, the control term would fall from 2.40 to 0.05 USD; both conventions are derived in File S6b. Table 3 gives every path as figures and Figure 7 places the same paths in cost–time space.

Three results in that table determine the architecture. Sequential testing would be expensive as well as slow, because a second run pays for a second control pair as well as a second 45 min block: 8.20 USD at 99 min against 10.60 USD at 144. The critical configuration has a break-even point, and M2 is what decides on which side of it a specimen falls. M2’s output is a probability *q* that a resistance determinant will need to be interrogated, and *q* enters the arithmetic directly rather than through a ranking: co-loading species now and escalating later costs 8.20 + 4.80*q* USD against a flat 10.60 for carrying the resistance positions from the start, so the two are equal at *q* = 0.5, above which the critical configuration is cheaper as well as faster. That is a decision made on the numeric value of a predicted probability, which is why calibration is a release criterion for M2 and not a reported statistic. The break-even itself is an accounting one over consumables: it contains no missed resistance determinant, no delay to targeted therapy and no patient harm, and the threshold at which resistance positions should be carried is a decision-analytic question with a different answer, almost certainly a lower one. What *q* = 0.5 establishes is that the reagent budget stops arguing against the critical configuration at that point, not that the clinic should wait until then.

Triage is worth more than it appears. If 40% of specimens genuinely require species identification, running the standard configuration on those and the kingdom-only configuration on the remaining 60% gives a mean consumable cost of 6.04 USD, against 8.20 USD for the standard configuration on everything—35.8% more reagent for no gain in turnaround whatsoever, because under the release rule every configuration takes the same 99 min. Running kingdom-only on everything and escalating reactively is the third strategy and costs 7.00 USD at a mean of 117 min, because the 40% that escalate wait for a second run. Triage-directed selection is therefore the only one of the three not dominated, and the accuracy it needs is modest: degrading M1 to a sensitivity of 0.80 and a specificity of 0.80 together changes the mean cost by 0.62 USD and the mean time by 3.6 min, which still leaves it cheaper than either strategy that does not triage. File S6d derives all four rows branch by branch: the three strategies above, and the degraded-M1 variant of the first, which is what the 0.62 USD is measured against rather than a fourth way of running the platform. The two ways of being wrong are not paid in the same currency, and both belong in the sensitivity analysis: a specimen M1 misses escalates to a second run, waiting 144 min instead of 99 and costing 2.40 USD more, while a specimen M1 over-triages loses no time at all and costs 3.60 USD more. An earlier version of this analysis degraded sensitivity alone, which assumes a specificity of one and so made the triage error free in reagent by construction.

What the unclosed channel budget would do to these costs and timings. Section 4.8 states that the channel budget is not closed: one detection channel in every reaction is now committed to the internal amplification control, and the reserved core block carries more distinguishable signals than the remaining channels hold. Three resolutions are open and they do not cost the same, so the consequence for the arithmetic above is stated here rather than left to be worked out. Adding channels to the instrument leaves every entry in Table 3 unchanged, because no position changes; what changes is the instrument specification, and with it the assumption recorded in Table S9 that a six-channel instrument suffices. Splitting the core block across an additional position costs 1.20 USD per specimen and changes nothing else in five of the six configurations of File S9b, which occupy three, four, four, five and six of the eight positions and therefore have a spare. The exception is the critical configuration, which already occupies all eight: there an added position requires either a larger housing or a second run, and a second run buys its own control pair and its own 45 min block, changing that path from 10.60 USD at 99 min to 13.00 USD at 144. That would remove the critical configuration’s advantage altogether. The break-even point above is 8.20 + 4.80*q* against a flat 10.60; against a flat 13.00 there is no value of *q* below one at which carrying the resistance positions from the start is cheaper. Accepting an indeterminate call in polymicrobial specimens is the third resolution and changes neither cost nor time; it is paid in diagnostic yield instead. Which resolution is chosen is therefore not only an assay decision. It determines whether the cost arithmetic of this section survives contact with the finished specification, and only the first of the three leaves it intact.

The comparison that matters is against the episode cost the test changes. In the INHALE WP3 randomised trial, in-ICU syndromic PCR gave mean ICU costs of £33,149 against £40,951 for standard care, and a Monte Carlo model of blood-culture identification panels found savings of roughly 8600 Canadian dollars per admission [130,131]. The clinical result of that trial has to be carried with its cost result. INHALE WP3 had two co-primary endpoints: superiority for antibiotic stewardship at 24 h, which it met, and non-inferiority for clinical cure of pneumonia at 14 days, which it did not demonstrate. A faster and cheaper answer that does not improve—or that worsens—cure is the outcome this section’s endpoint hierarchy exists to prevent, and it is the strongest published argument against the proposal made here. It is the reason clinical utility, and not stewardship or turnaround, is the endpoint named in Section 4.1. Against differences of that magnitude, a single-digit consumable cost is not decisive, but it decides whether the test can run at all where a cartridge-priced platform cannot. Two of the four sustainability dimensions set out in Section 1 are settled here, both as metrics to be measured rather than properties held. Resource intensity is reagent cost per actionable result, against the published cost-effectiveness of rapid molecular resistance detection [132], with the 43 min difference between the robot-assisted and manual routes counted as technologist time released rather than eliminated. Environmental footprint matters because healthcare accounts for approximately 4–5% of global greenhouse-gas emissions [133]; the design is projected to remove plates, broths and incubation energy, to avoid the second run, and to remove refrigerated storage from the supply chain, the last resting on stability data that do not yet exist. The metric is grams of plastic and kilowatt-hours per actionable result, and none of it is measured. Antimicrobial sparing and deployability belong to where the platform is put rather than to the run, and are taken up in Section 5.4.

### 4.7. How the Platform Addresses the Failures Identified in the Review

Each failure Section 3 measured has a design response, and Supplementary File S19 sets them out item by item with the frequency of each failure in the appraised corpus. Read down that table, the responses have one thing in common: each constrains what a model is allowed to do rather than improving how well it does it—external evaluation as the primary design rather than an internal split, calibration as a release criterion rather than a reported statistic, a sample size computed in advance under the Riley framework [25], published coefficients and thresholds, pre-specified subgroup analysis, a registered protocol, and continuous drift monitoring against incoming culture results, following the one prospective design in the corpus that measured decay [12]. That is the shape of the answer this review implies. The same point is placed on the working sequence in File S18, with the seven model decisions above the line and the decisions that remain a person’s below it.

### 4.8. Limitations and Staged Deployment

Seven limitations determine what must be demonstrated, and in what order; Supplementary File S22 turns them into an analytical validation plan with acceptance criteria fixed in advance.

Analytical sensitivity in blood is the binding constraint. Most episodes of clinically significant bacteraemia in adults carry one to ten colony-forming units per millilitre [134], which may sit below the limit of detection of a small-volume assay run without enrichment, and no model compensates for a target that was never amplified. Sepsis therefore carries the greatest clinical value and the hardest validation, and must come last; urine and stool first, respiratory specimens next; and hospital laboratory first and primary care last, because the operator, the quality-control regime and the safe-failure behaviour of the report all need demonstrating before a test that withholds antibiotics is placed outside a laboratory.

The releaser carries the largest unproven assumption. That a single lysis step recovers an amplifiable template at an efficiency comparable to column purification while depleting the human background is what removes purification and compresses the analytical phase. Every timing figure above is downstream of it, and it is a premise rather than a result—weighing most on the protozoal channel, whose 18S is close enough to ours that host depletion is doing the discriminating rather than assisting it. Recovery against a column reference, per specimen type, is the first study required.

The combined reaction is a hypothesis, and multiplexing has a sensitivity price the channel count conceals. At least six primer pairs compete for one polymerase—six optical channels are read, and a channel may carry more than one pair, so the number of primers in a tube is a floor set by the channel count rather than a ceiling—and the competition is worst where it matters most: a high-copy host cellularity control shares the reaction with a pathogen target present at a few copies, and reading all six channels as targets leaves the reaction without its own amplification control. Placing the internal amplification control in the group-determination position, as an earlier version of this specification did, makes a reflex second run inherit its inhibition evidence from a reaction it does not share: the targeted resistance and species configurations carry no group tube, and a second run has its own pipetting, its own reagent positions, its own multiplex competition and its own lyophilisation lot. A negative result from it would then rest on evidence collected before any of those existed. That inheritance is withdrawn. Inhibition is a property of a reaction and must be measured in one, which is also what File S22 requires of the validation—per position, not per specimen—so the two control positions every configuration in File S9b already carries are specified as a no-template control and a combined positive and internal amplification control, in the second-run configurations as much as the first. To be explicit, because the two sit at different levels: the control positions validate the strip and its lyophilisation lot, while inhibition in a given specimen reaction is reported by the internal amplification control co-amplified inside that reaction. A control in its own well cannot report inhibition in a well it does not share. This is a requirement placed on the design, not a solved problem: it commits one detection channel in every reaction to the control, and the reserved core block described in Section 4.4 is already the tightest constraint on the channel budget. The channel budget is where these commitments collide, and it is not yet closed. *Staphylococcus aureus* with *mecA* and *mecC*, Enterobacterales with the common carbapenemase determinants and *Legionella pneumophila* are more distinguishable signals than six channels hold, and multiplexing several into one channel would lose exactly the information a polymicrobial specimen needs, namely which organism carries which determinant. Whether the core is delivered by more channels, by splitting it across positions, or by accepting an indeterminate call in polymicrobial specimens is a design decision this projection does not make, and a per-target channel and position map is the first thing a real specification would have to publish. Beyond that, the assay is not specified to a level at which anyone could reproduce it. Missing are the probe chemistries and fluorophore–quencher pairs, primer and probe concentrations, amplicon lengths matched across channels, the cycling protocol, inclusivity and exclusivity panels, cross-reactivity against the commensal flora of each route, multiplex interference target by target, and—for the respiratory RNA viruses entering through the reserved channel—a reverse-transcription step the workflow does not specify at all. Until those exist, every figure here describes an intended assay rather than a characterised one.

Reagent-borne contamination survives lyophilisation. The “kitome” of *Ralstonia*, *Bradyrhizobium*, *Delftia* and *Cutibacterium* sequences carried in polymerase reagents is a reproducible artefact, and it most affects the low-biomass sterile-site specimens for which broad-range detection is most wanted. The closed, pre-dried format is projected to remove operator-introduced, master-mix and cross-sample contamination, but not contamination lyophilised in, and the size of that reduction is a measurement nobody has made for this format. Lot-matched no-template and blank controls are therefore a release criterion rather than good practice, and the release rule of Section 4.5 rests on them.

Genotype is not phenotype. Absence of a known gene does not exclude resistance arising through efflux, porin loss or regulatory mutation, and presence does not guarantee expression at a level causing clinical failure; the genomic domain shows models falling from AUC values above 0.93 internally to approximately 0.77 on independent data [37], and breakpoints themselves are versioned [100]. Determinants must be reported as evidence that shifts a probability, never in the visual grammar of a susceptibility report, and phenotypic testing runs in parallel.

The models learn from prescribing, not from correctness. A model trained on what clinicians prescribed learns habit, including over-broad empirical cover; one appraised study predicts the therapy given rather than the therapy that proved appropriate [74]. The recommendation modules must be trained against an adjudicated appropriateness label defined by the eventual susceptibility result, and must report inadequate recommendations separately from unnecessarily broad ones—the error that harms patients apart from the error that drives resistance.

The regulatory path belongs in the development plan, not after it. The assay and the software follow different routes: the assay is an in vitro diagnostic requiring a performance evaluation, while modules recommending an agent (M3) or a dose (M4) are software as a medical device, and a device that withholds or narrows antimicrobial therapy will not sit in the lowest risk class. Which rule and class each falls under depends on the stated intended purpose and needs a regulatory specialist rather than this paper; what the paper does claim is that classification, a clinical performance study and post-market follow-up have to be specified alongside the analytical work, because a programme treating approval as a downstream step will find it collected the right evidence in the wrong form.

Within these limits, the *Clostridioides difficile* infection (CDI) and microbiota-restoration module warrants an explicit boundary. Restoration for recurrent *C. difficile* infection is guideline-directed therapy with cure rates approaching 90%, but the decision to administer belongs to a clinician: the module stratifies risk and flags eligibility for review, and the platform should not be built so that it could do more [135,136,137]. Raising the question when the antibiotic is prescribed rather than after a recurrence has two grounds. Restoration acts on the reservoir rather than the episode [138] and it should be an ecosystem rather than a defined product, since resistance determinants are widespread among probiotic bacteria and the gut is a site of horizontal transfer, so defined strains added to a gut still holding residual pathogens supply a gene donor to the organisms one is trying to displace [139,140,141]. Autologous material must therefore be taken before or with the first dose. Restoration after a routine course in a patient who has not had *C. difficile* infection is an investigational use with no guideline support, offered as a design position rather than a recommendation.

The order in which these would have to be answered. The limitations above are not a list of caveats but a sequence, and stating it is the point of the section: a proposal whose falsification path is not specified is not a testable one. Stage 1, analytical validity, on contrived and residual specimens, includes the limit of detection to 95% probability per target and per matrix; inclusivity across the strain panel and exclusivity against the commensal flora of each route; extraction recovery against a column reference; inhibition per position, with the internal amplification control of Section 4.8; multiplex interference target by target; carry-over; and lot-to-lot and operator-to-operator precision. Nothing beyond this stage is meaningful until the assay is characterised against the acceptance criteria set out in File S22. That file is also explicit about what it does not yet fix, and the gap belongs in the main text rather than in a supplement: the numerical boundary of the declared indeterminate state, the control-failure rules—including the number that makes a no-template control “clean” at the end of a run, on which the release rule of Section 4.5 turns—and the reference materials that would make a limit of detection in copies per millilitre traceable, and reporting of the assay itself compliant with the Minimum Information for Publication of Quantitative Real-Time PCR Experiments (MIQE) guidelines. Those four must be pre-specified before the first study in this stage is run, and none of them exists today. A fifth gap is not S22’s but this design’s own. File S7 makes a lot-matched extraction blank mandatory alongside the no-template control wherever a broad-range 16S reaction is read on a low-biomass specimen, and the strip does not carry one: its two control positions are a no-template control and a combined positive and internal amplification control, and those two are what Table 3 prices. A blank taken through the lysis and releaser step is not the same control as a no-template control added at the reaction, and in cerebrospinal fluid, a joint aspirate or tumour tissue, it is the one that decides whether a positive belongs to the patient or to the reagent lot. Whether it is carried as a third control position, as a separate run, or as a per-lot qualification performed away from the instrument changes both the cost per specimen and the throughput, and this projection does not settle it. Stage 2, clinical validity, prospective and multi-site, includes agreement against culture and susceptibility testing as the reference standard, sized in advance, with calibration reported as a primary endpoint for every module whose output crosses a threshold, and performance reported separately across sites and across patient characteristics rather than pooled. This is the stage at which each of M0 to M6 becomes a locked model with a published specification under TRIPOD+AI, and at which the six-channel and control-position questions of this section are resolved by measurement rather than by argument. Stage 3, clinical utility, against usual care, includes time to appropriate therapy, duration of unnecessary broad-spectrum exposure, and patient outcome, with the reflex-trigger policy of Section 4.5 and the *q* threshold of Section 4.6 re-derived from observed consequences rather than from consumable cost. Failure at any stage stops the next, and the honest statement of where this proposal now sits is that not one item of Stage 1 has been performed.

## 5. Discussion

### 5.1. Principal Findings

Ten years of work have produced a large, fast-growing and technically competent literature on artificial intelligence and antimicrobial resistance, and very little clinically deployable evidence. The gap is not in whether discrimination is reported, which it is in every study appraised; this review did not extract performance values and so says nothing about how good they are. It is in everything that would allow a clinician to rely on that performance: external evaluation in 45% of appraised studies, calibration in 23%, sample-size justification in 16%, a model a reader could apply in 24%, and a comparison of performance across patient characteristics, as opposed to across study sites, in five. Where external evaluation was done it was done properly—in 31 of those 36 the evaluation data came from another institution or a separately assembled dataset rather than from a later period at the same one—so the shortfall is in how many studies validate externally, not in how weakly they do it.

The most actionable finding is the misuse of the term external validation or independent in 8.8% of appraised studies, with a further study calling an 80/20 split a validation across four species. This is a correctable problem, and it is correctable at the level of peer review: a reviewer who asks a single question—from what population, collected when and where, did the evaluation data come?—would catch every instance we identified. Its persistence indicates that the term has drifted in this literature to mean “data not used to fit the final model”, which is a description of a test split, not of external validation.

### 5.2. Comparison with the Wider Methodological Literature

The pattern found here is not specific to antimicrobial resistance. Systematic appraisals of clinical prediction models in other fields have repeatedly reported high risk of bias concentrated in the analysis domain, sparse calibration reporting and rare external validation, which is precisely why PROBAST+AI and TRIPOD+AI were developed [14,15]. What may be specific to this field is the outcome-definition problem: susceptibility is defined by a versioned breakpoint standard, and a study that does not state which version it used has not fully specified its label. Twenty-six of the 77 studies to which the development pass applies (33.8%) were rated unclear on the outcome domain, most of them for this reason.

These findings are not isolated, and the agreement is informative. Three prior systematic reviews that appraised prediction models for multidrug-resistant organisms with PROBAST reached the same qualitative verdict—high risk of bias concentrated in the statistical analysis, external validation in a minority, and calibration that was seldom reported [17,18,19]. That those reviews drew on different studies under different eligibility rules argues that what we observe is a property of the field rather than of our sample. It also sharpens the one place where our reading departs from theirs. Where a prior review counts external validations as reported—fewer than a quarter of models, in the largest of them—we read each study’s description of its design against what the study actually did, and found 8.8% applying the term to a random split of a single dataset. A pooled estimate built on the label rather than on the procedure is therefore an upper bound, and the gap between the two is itself the finding.

A second field-specific issue is that the genotype-to-phenotype relationship is imperfect in a way that a performance metric conceals. Absence of a known resistance gene does not exclude resistance arising through efflux, porin loss or regulatory change, and presence does not guarantee clinically relevant expression. The external-validation performance drops observed in the genomic domain are, in part, this biological gap becoming visible when the population changes.

Three of these findings are new to this literature rather than confirmations of it, and it is worth separating them from the agreement described in Section 5.2. The first is that the shortfall is common to all four domains. Genomic prediction, clinical risk models, therapy and dosing, and rapid diagnostics are reviewed apart from one another and differ in almost every other respect; read under one rule they fail on the same four items, which makes the deficit a property of the field rather than of any one application. The second comes from the appraisal instruments themselves, which ask questions their predecessors do not: a model a reader could actually apply, present in 24%, and performance compared across patient characteristics, present in five of eighty, are reported by none of the reviews in Table 1, because none of them had an instrument that asked. The third is the distance between what a study calls its validation design and what it did. That cannot be established from an abstract, and no earlier appraisal we identified has measured it, and at 8.8% it means that any pooled estimate built on the label rather than on the procedure is an upper bound. Table 1 places each of these against what the previous reviews established.

### 5.3. Implications for Practice

Three implications follow for anyone considering deploying such a model.

First, a published AUC should be treated as an upper bound obtained under favourable conditions, not as an expected operating characteristic. The observed drops on genuinely external data—from above 0.93 to approximately 0.77 in one well-conducted example [37]—are large enough to change a clinical decision rule.

Second, a model without reported calibration cannot support a decision that depends on the numeric value of the predicted probability—panel selection, treatment escalation and stewardship triggers are all of that kind, and the *q* = 0.5 break-even point of Section 4.6 is exactly such a decision. That is a narrower statement than unusable: a model that discriminates well but is uncalibrated can still be deployed at a fixed operating point whose sensitivity and specificity were established on external data, and can still rank patients for review. What it cannot support is a threshold chosen on the assumption that a predicted 0.3 means a thirty-per-cent risk.

Third, performance decays. The single study that measured drift prospectively found it [12]. Any deployment without continuous monitoring against incoming culture results should be regarded as unmonitored, not as stable.

### 5.4. Implications for the Platform Proposed Here

The platform specified in Section 4 was designed against these findings rather than around them, and its central commitments are direct responses: prospective multi-site evaluation as the primary design, calibration as a primary endpoint because panel selection is threshold-driven, sample size computed in advance, models published in usable form, subgroup performance analysed as a pre-specified rather than exploratory analysis, and continuous drift monitoring.

Two of the four sustainability dimensions set out in Section 1—antimicrobial, economic, environmental and geographic—are properties of where the platform is placed rather than of the run, and both are stated as endpoints to be measured rather than as achievements. Antimicrobial sparing is the primary argument and an ecological rather than an economic one: an organism-directed first dose reduces the broad-spectrum therapy given for coverage rather than for evidence, and with it the selection pressure that generates the next resistant generation. The endpoint is days of therapy per 1000 patient days by World Health Organization AWaRe category, with the Watch and Reserve fraction primary, against empirical prescribing at the same institution over the preceding period [142]. Deployability follows from a workflow that needs one instrument, no culture facility and, if the lyophilised format performs as designed, no cold chain; that combination opens a tier culture-dependent diagnostics cannot reach—primary care, where most antimicrobial consumption in the European Union and European Economic Area occurs, rather than in hospitals [143], and where point-of-care testing already changes prescribing for a far cruder marker, C-reactive protein [144,145]. It is also where the evidence is thinnest: health inequalities were mentioned in 3.8% of the appraised studies, and one of the corpus’s few external validations, an England-to-Africa transfer, showed exactly the performance loss that going unmeasured would have concealed [8]. That is why primary care is the last deployment tier in Section 4.8 despite carrying the largest prescribing volume, and why performance stratified by site resource level has to be a primary analysis rather than a subgroup afterthought.

Two design decisions deserve restating because they are where the sustainability argument and the safety argument meet. Adaptive panel selection is what makes the workflow resource-efficient, and it is also what creates the risk of missing an organism that was never tested for; the response is a physically reserved block of core targets in every manufactured strip, a one-way escalation rule, and a report format that names the targets that were not tested. The lyophilised closed-tube format is what makes the workflow deployable without a cold chain, and it is also what structurally reduces the contamination risk that is the standing objection to culture-independent PCR.

### 5.5. Strengths and Limitations

The main strengths of this review are that the search was retrieved to exhaustion rather than sampled, that the search log was written at search time, that every screening decision is recorded with a reason, and that the appraisal items on which the central claims rest were human-coded from full text after automated coding was shown to be unreliable for exactly those items. Every reference was audited on seven dimensions—DOI, first author, full author list, journal, volume and issue, pages or article number, and PMID—each checked against a fresh PubMed record and against the Crossref record, with the differences that are database formatting rather than error separated from the ones that are not (Table S12). The audit was re-run over the printed list of this revision, so that it covers the four references added here as well: 144 of the 145 entries carry no discrepancy on any dimension, and the one that does is a disagreement between the two authorities rather than an error in the entry—PubMed records the DOI suffix of reference [94] as its page, where the publisher’s own citation metadata and Crossref both give pages 1–14. The per-entry result is in File S12b.

Several limitations should be weighed, and all of them are itemised against PRISMA 2020 in Supplementary File S17. The search covered PubMed only; Embase, Scopus, Web of Science and the grey literature were not searched, no citation chasing was performed, and conference abstracts and preprints were excluded by design, so the conference literature enters only through the small number of full proceedings papers that PubMed indexes. This is the review’s largest coverage limitation, and two consequences follow. Methodological work published at machine-learning venues rather than in indexed journals is missing altogether, which means the genomic-prediction domain in particular is described here by its clinical-journal fraction rather than by the whole of it; where that literature is methodologically stronger, the review understates the field, and where it is weaker—smaller samples, no clinical reference standard—it overstates it, and we cannot say which dominates. And because the eligible set is what the temporal and domain comparisons are computed over, those comparisons describe the PubMed-indexed literature and should not be read as describing the entire field. Early stage work is under-represented for the same reasons. Screening and appraisal were performed by a single reviewer, without duplicate independent assessment, which is a departure from systematic-review standards and may introduce inconsistency; the full decision record is provided so that the judgments can be inspected and challenged. Because that is the review’s central weakness, we quantified it rather than only declaring it. Sixteen of the eighty appraised studies, drawn at random under a stated seed, were re-coded from full text by a large language-model agent given the coding rules and the papers but no sight of the original records, and the two codings were compared item by item (Supplementary Table S16). This is not a second human reviewer and is not offered as one; what it establishes is which items are robust to who is reading. Agreement was complete for calibration (16 of 16) and high for study type (15 of 16), for the accuracy of the authors’ own description of their validation design (14 of 16) and for the validation design itself (13 of 16). It was substantially lower for sample-size justification (10 of 16) and lowest for the overall PROBAST+AI risk-of-bias judgment (7 of 16). The verification pass described in Section 2.6 extends that check from sixteen studies to all eighty on the four items that carry the review’s conclusions. Of 320 comparisons, 297 agreed (92.8%): 76 of 80 on validation design (Cohen’s kappa 0.90), 72 of 80 on the accuracy of the authors’ own description of it (0.54), 72 of 80 on calibration (0.74) and 77 of 80 on subgroup performance (0.86). Every one of the 23 disagreements was adjudicated in writing against the full text, the first coding being overturned in 16 and the second in 7, and all 320 comparisons are given with their quotations in Supplementary Table S16b. Because the coder had already seen the original code of 39 studies, agreement is reported separately for the two halves: 93.3% where the code had not been seen (153 of 164) and 92.3% where it had (144 of 156), so the partial unblinding did not inflate it. The item with the weakest agreement is the one the review’s most actionable claim rests on—whether an author’s own description of their validation design is accurate—and a kappa of 0.54 is the honest measure of how much judgment that reading requires. Section 3.4 therefore reports that claim as the range the three codings span—eight of 80 after adjudication, and nine and 11 under each coder alone—rather than as a point estimate. This remains a machine re-coding rather than a human duplicate, and the reliability of a second human reviewer is not something either it or the sixteen-study sample can estimate. The risk-of-bias disagreements were almost all in one direction: in eight of the nine, the second coding rated high where the original rated unclear. The boundary between those two categories is a matter of how much benefit of the doubt a reader extends to an incompletely reported analysis, and our judgments sat on the generous side of it. Readers should therefore treat the distribution in Section 3.5—low in 16, unclear in 55, and high in nine—as a conservative one, and the proportion at high risk of bias as more likely to be understated than overstated. The core set of 80 is a purposive, priority-weighted sample rather than a random one, and was deliberately weighted towards externally validated and clinically evaluated work—which means the reported figures for external validation, calibration and sample-size justification are, if anything, more favourable than the field as a whole. Requiring a retrievable full text may have introduced a further selection effect. The abstract-level evidence map understates reporting, and is presented only as a lower bound and as a measure of what authors foreground. Finally, PROBAST+AI judgments were recorded at the domain level with written rationales rather than as a published answer to each of the 16 development and 18 evaluation signalling questions; the underlying facts are provided per study, but readers wanting item-level judgments will not find them here.

We are not the first to appraise prediction models in this field with a formal risk-of-bias instrument, and where our conclusions overlap with earlier reviews we say so in Section 5.2; what is claimed here is the breadth of the sample, the extended instruments and the validation-terminology result, not the discovery that the evidence base is weak.

It is worth holding this review to its own standard, since the shortfalls it reports in the corpus are ones it partly shares. It was not registered and no protocol was written before the work began—the same two omissions found in 93% and 99% of the appraised studies. Screening and appraisal were performed once rather than in duplicate by two humans. The core set was fixed at twenty per domain for tractability rather than derived from a target precision. No reporting-bias assessment and no certainty-of-evidence grading were performed. File S17 records all six against the PRISMA 2020 checklist, and separates, item by item, a failure to report from a limitation of method—the single-reviewer design is fully reported, which is what PRISMA items 8, 9 and 11 ask, and is a weakness of method rather than of reporting. We report them because a review that asks the field for pre-specification and independent evaluation has no standing to omit its own account of them.

The platform described in Section 4 is a proposed architecture. It has not been built, and no analytical or clinical performance data are presented for it. The time budget is a step-by-step calculation from a stated instrument specification, not a measurement.

## 6. Conclusions

This review began with a mechanism rather than a technology: a patient presents, the organism is unknown, and for the twenty-four to seventy-two hours it takes to name it the clinician prescribes for coverage rather than for evidence. Every hour of that uncertainty is paid for in selection pressure, and it is paid whether or not anyone is watching. Artificial intelligence can plausibly shorten the interval between a patient presenting with an infection and receiving the right antibiotic, and shortening that interval is one of the few interventions that reduces selection pressure without asking clinicians to accept more risk. The evidence assembled here shows that the field has demonstrated the capability many times over and has almost never demonstrated the reliability. External validation in 45% of the appraised studies, calibration in 23%, sample-size justification in 16% and a patient-subgroup performance analysis in five describe a literature optimised for publication rather than for deployment.

The remedy is not more models. It is a shift in what counts as a complete study: prospective evaluation outside the development population, calibration reported as a matter of course, a model that someone else can actually run, performance examined across the subgroups that the intervention will reach, and monitoring that continues after deployment. None of this requires new methods; all of it requires convention to change.

Sustainability should be held to the same standard. A diagnostic that improves antibiotic selection while increasing reagent consumption, cost, waste and cold-chain dependence has changed the problem rather than solved it. Designing for antimicrobial sparing, resource intensity, environmental footprint and deployability in low-resource settings from the outset—and measuring each of them—is what distinguishes a sustainable approach from a faster one.

What we have described should be read as a template rather than a finished product, and its adaptability is a design property rather than a concession. Resistance is local and it changes; a platform whose targets are fixed at manufacture is obsolete on a schedule set by the organisms. Four things in this architecture are therefore deliberately variable. The strip library is reconfigurable, because the targets sit in lyophilised tubes rather than in the instrument, so a site with a carbapenemase problem and a site with a fluoroquinolone problem run the same hardware with different strips. The model layer is retrainable on local data, which matters most for the two modules whose probabilities enter arithmetic directly, since M1’s group probabilities and M2’s *q* are only meaningful against the epidemiology they were calibrated on. The deployment tier is a choice and not a prerequisite: the same closed-tube, room-temperature format that suits a hospital laboratory is what makes a decentralised laboratory and, eventually, primary care reachable, and it is in primary care that most prescribing happens. And the kingdom-first logic is not specific to the syndromes discussed here—the question of which kingdom is causing this illness precedes every other question in any infection, which is what allows the same architecture to be pointed at a different clinical problem without being rebuilt.

Calling this a testable model is a commitment rather than a description of work already done. Every parameter that drives the figures in Section 4 is tabulated with its provenance, each classified as measured, vendor-specified, a design decision or an assumption (Table S9), and each of the seven modules is specified with its model class, inputs, output, calibration requirement, validation design, primary failure mode, safeguard, the decision that stays human and the decision it replaces (Table S10). That is enough for a group other than ours to price the consumables against their own costs, run the time budget against a clock and falsify the numbers claimed here. It is not enough to build the assay: Section 4.8 lists what the specification still lacks—the probe chemistries, the primer and probe concentrations, the cycling protocol and the reverse-transcription step among them—and completing it is work, not transcription. The study that should follow is therefore the Stage 1 analytical characterisation of Section 4.8, not the clinical comparison, because this design’s own rule is that failure at any stage stops the next and not one item of Stage 1 has been performed. The prospective, multi-site comparison of time to first appropriate therapy against the local standard pathway is the endpoint that would settle whether the platform is worth having, and it belongs at the end of that sequence rather than at the start. Until it exists, nothing in Section 4 is evidence.

What follows from this, concretely. Four things are now specified well enough to be done, and they are given in the order their dependencies impose. First, the four rules that File S22 leaves open—the numerical boundary of the indeterminate state, the control-failure rules including what makes a no-template control clean at the end of a run, the reference materials that would make a limit of detection traceable, and MIQE-compliant reporting of the assay—have to be fixed in writing before any wet work, because each of them determines how the first experiment would be read. Second is the Stage 1 analytical characterisation of Section 4.8—limit of detection per target and per matrix, inclusivity and exclusivity, extraction recovery against a column reference, inhibition by specimen type, multiplex interference, carry-over, and lot and operator precision, against the acceptance criteria in File S22 and including the lot-matched extraction blank that the present strip layout does not carry. Third, and only then, is the prospective multi-site comparison, sized in advance, with calibration as a primary endpoint and time to first appropriate therapy as the clinical one. Fourth, and independently of whether the platform is ever built, is whether the review’s own finding is actionable at the point of peer review: a single question—from what population, collected when and where, did the evaluation data come?—would have caught every misuse of the term external validation identified here, and costs a reviewer one sentence. Each of the first three has an outcome that would falsify what precedes it, and failure at any of them stops the next.

The proposal is therefore modest in one respect and demanding in another. It asks for no method that does not already exist. It asks that the hour of diagnostic uncertainty with which this review opened be treated as a quantity to be measured, budgeted and reduced, and that any system claiming to reduce it be able to show—prospectively, with calibration, externally, and in the subgroups it will actually reach—that it does.

## Figures and Tables

**Figure 1:**
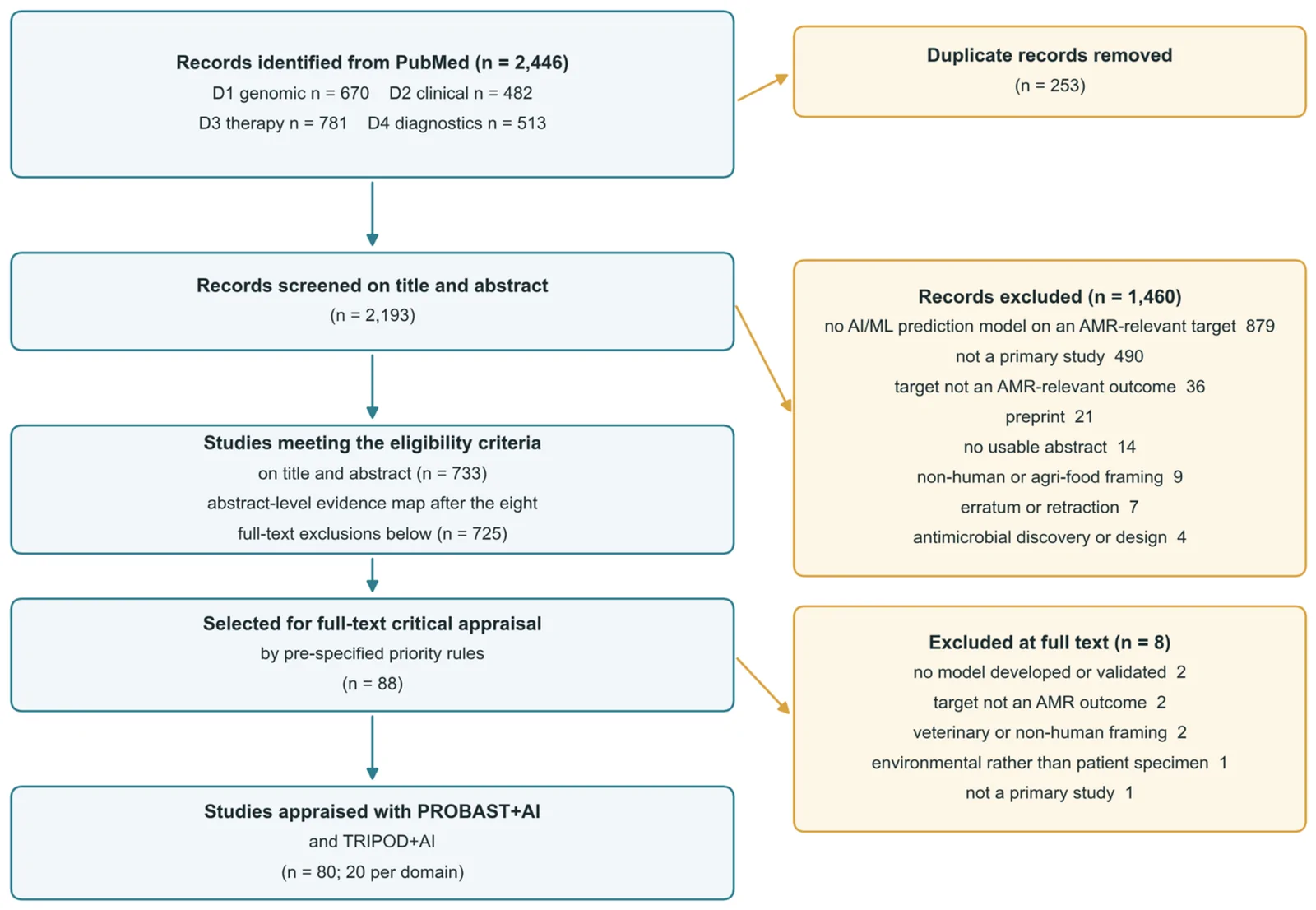
PRISMA 2020 flow diagram. The diagram is used here to report the search transparently, not to claim systematic-review status (Section 2.1); File S17 answers every checklist item, including the six shortfalls the review declares, which occupy seven No rows for the reason the file gives. The two-tier design is shown explicitly: 733 records met the eligibility criteria on title and abstract, of which 725 remained in the abstract-level evidence map after the eight full-text exclusions, and 80 of the 88 selected by pre-specified priority rules were appraised with PROBAST+AI and TRIPOD+AI. The record counts and the invariants they satisfy are in Supplementary File S1b.

**Figure 2:**
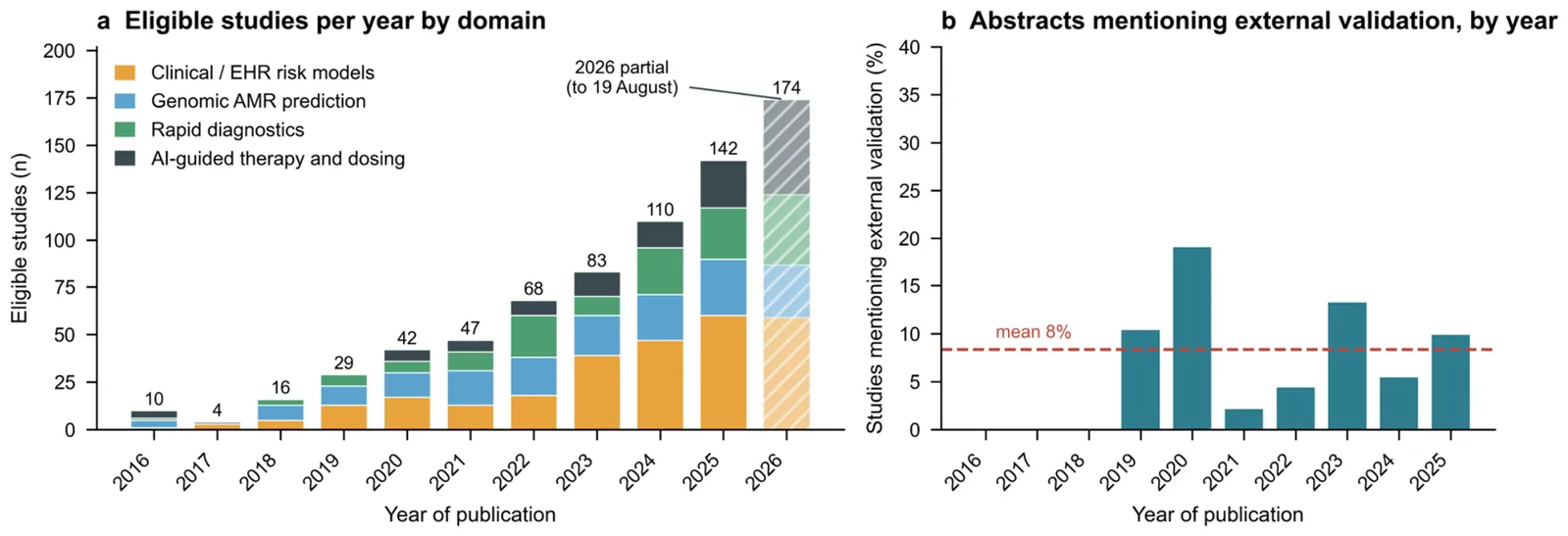
(**a**) Eligible studies per year by domain, 2016–2026. The 2026 bar is hatched because that year is complete only to the search date of 19 August 2026; it is shown for context and excluded from the primary trend analyses, for the reason given in Section 2.7; it is retained only in the two sensitivity fits reported in Section 3.1. Growth is steepest in the clinical and electronic health record domain. (**b**) The percentage of eligible studies whose abstract mentions external validation, by year of publication, over the complete years 2016–2025 only (n = 551). The dashed reference line is the mean over those years, 8.3%; the 12.0% quoted in Section 3.2 is the same quantity computed over all 725 studies, including the partial year 2026, in which the mention rate is 23.6%. The panel is plotted from the abstract-level indicator and therefore shows the use of the term, not the verified performance of the procedure; Section 3.4 separates the two.

**Figure 3:**
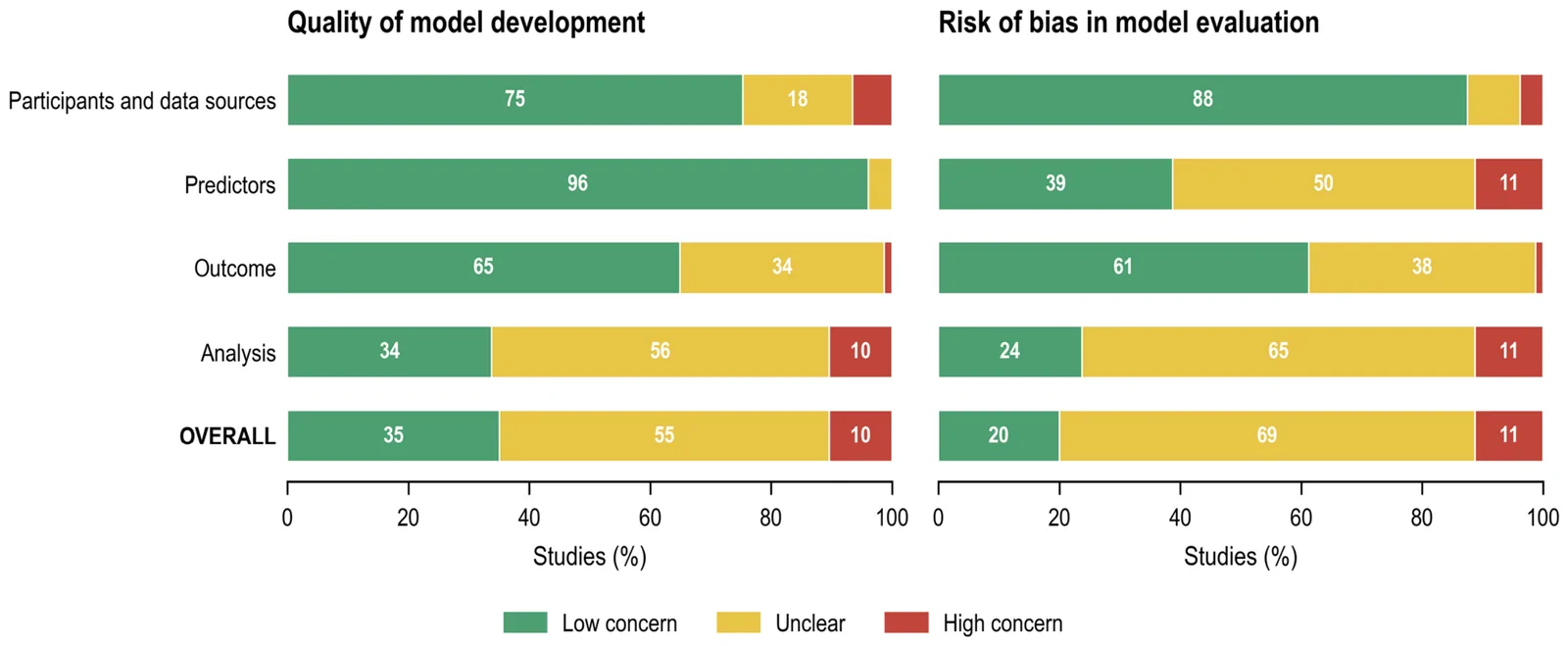
PROBAST+AI domain and overall judgments across the 80 appraised studies, shown separately for the quality of model development and the risk of bias in model evaluation. Concern is concentrated in domain 4 (analysis) in both passes.

**Figure 4:**
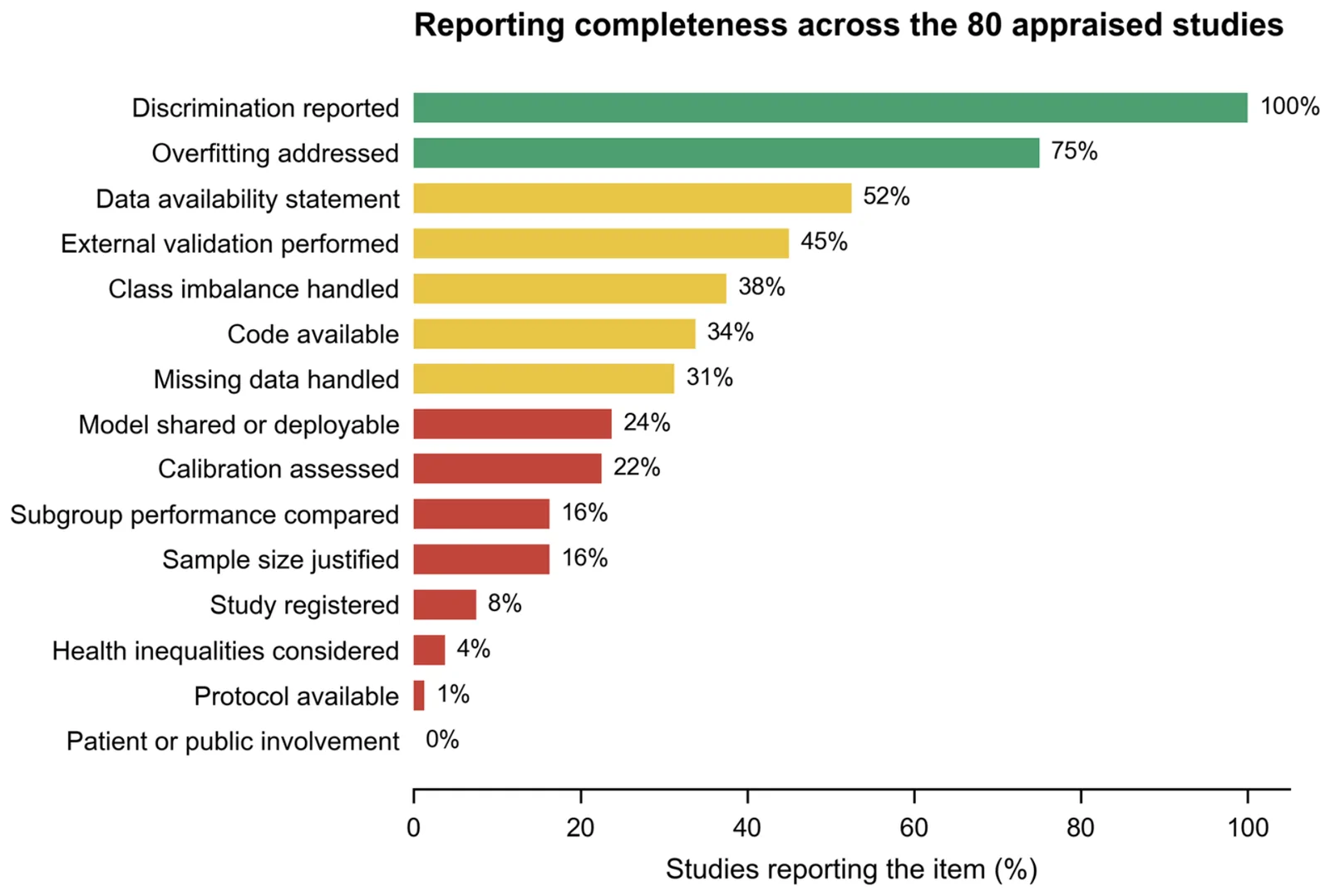
Reporting completeness across the 80 appraised studies. Items are ordered by frequency. Discrimination is universal and overfitting control common; everything that would let a reader judge, reuse or audit a model—calibration, the model itself, a sample-size justification, a comparison across subgroups, a registration and a protocol—sits in the lower half. The subgroup bar is any grouping, which Section 3.6 then splits into patient characteristics and study sites. Bars are coloured by the value they carry—red below 25%, amber from 25% to 60%, green at 60% and above—as a reading aid; the colour encodes no information the bar length does not.

**Figure 5:**
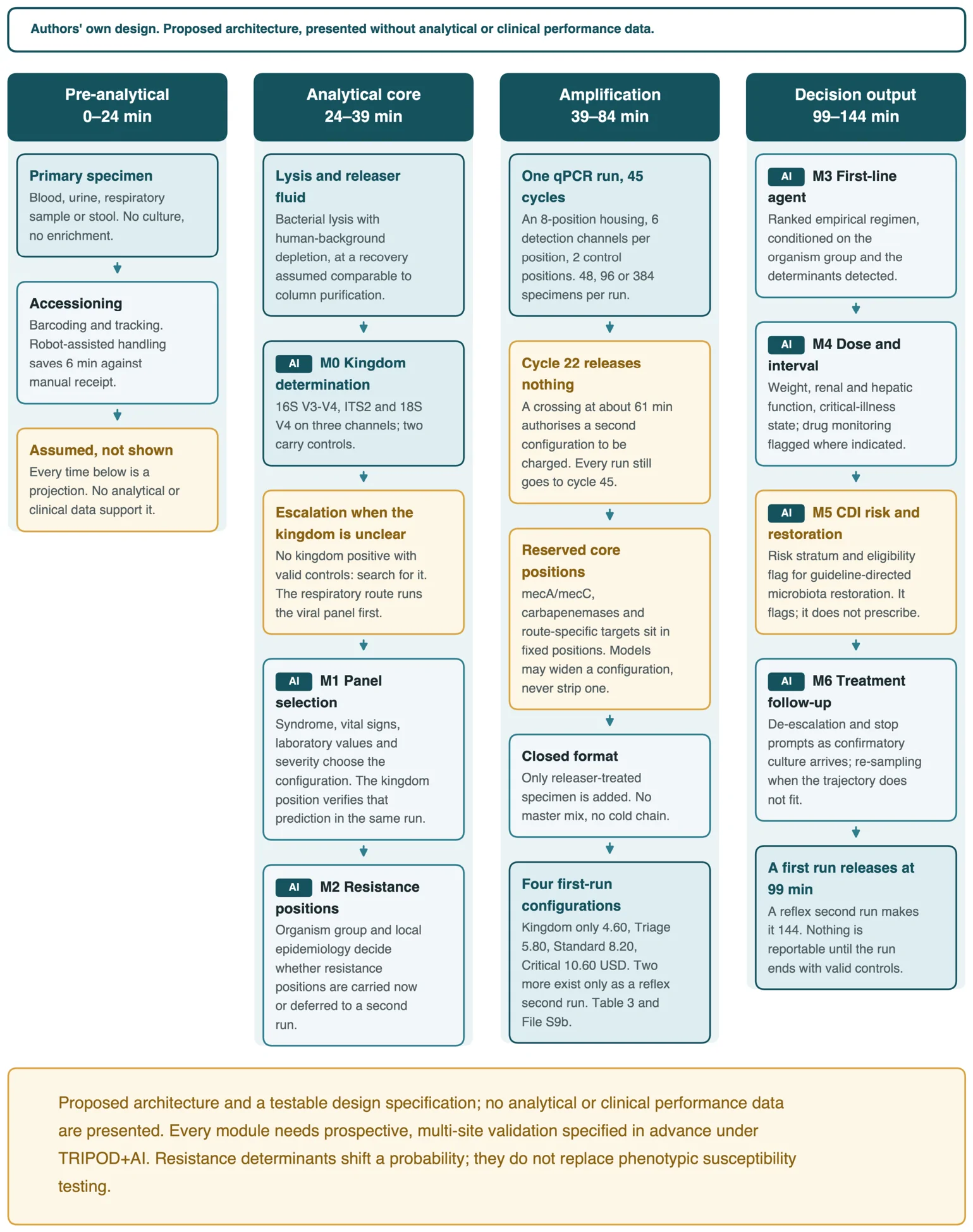
Architecture of the proposed platform: the authors’ own design specification, presented without analytical or clinical performance data of any kind. The four columns are the stages of one specimen’s passage, and the times in each header are the robot-assisted route of Section 4.5, projected rather than measured. Boxes carrying an AI chip are the seven model decisions M0 to M6; every other box is a wet-laboratory or handling step. Amber boxes mark the constraints that cannot be traded away—that every time in the figure is a projection, the escalation path when the kingdom call is ambiguous, the reserved core positions the models may widen around but never remove, the fact that a threshold crossing at cycle 22 authorises a second configuration and releases nothing, and the boundary on the *C. difficile* module, which flags eligibility but does not prescribe. Four configurations are selectable as a first run and two exist only as a reflex second; all six are set out with their contents and prices in File S9b, and the prices shown here are those of Table 3, derived from the same configuration object rather than transcribed. Three details that the earlier version of this figure carried as body text belong in the text and not in a panel: the kingdom-determination chemistry is given in Section 4.3 and File S7, the lysis and depletion assumption in Section 4.8, and the reasoning behind deferring resistance positions to a second run in Section 4.6.

**Figure 6:**
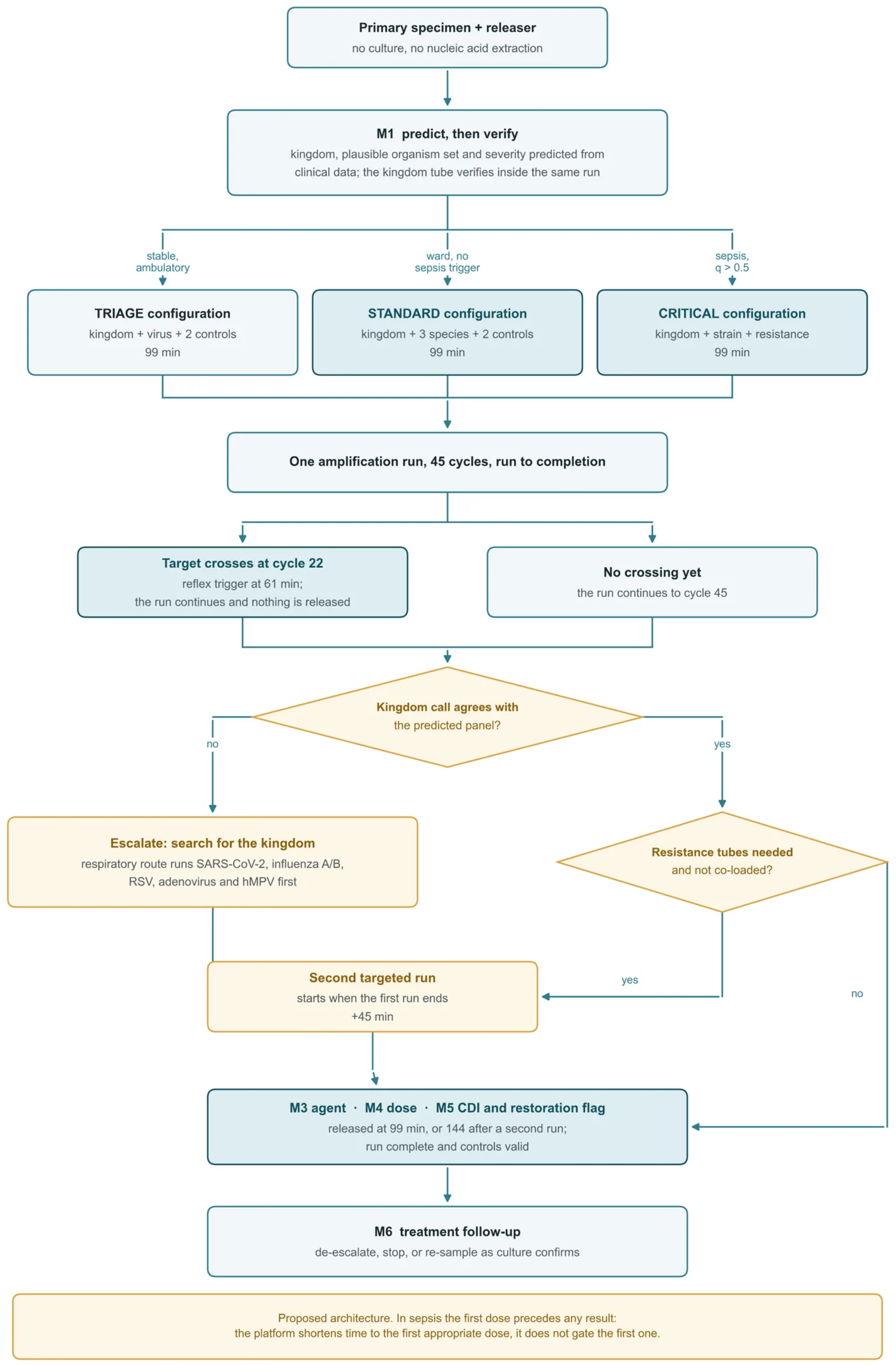
Decision flow from specimen to released result. M1 predicts the kingdom and the plausible organism set from clinical data, severity among its inputs rather than its outputs, and chooses one of the manufactured configurations available as a first run; the kingdom position verifies that prediction inside the same run rather than gating it. Every run is carried to completion: the crossing at cycle 22 marked on the diagram triggers the next step and releases nothing.

**Figure 7:**
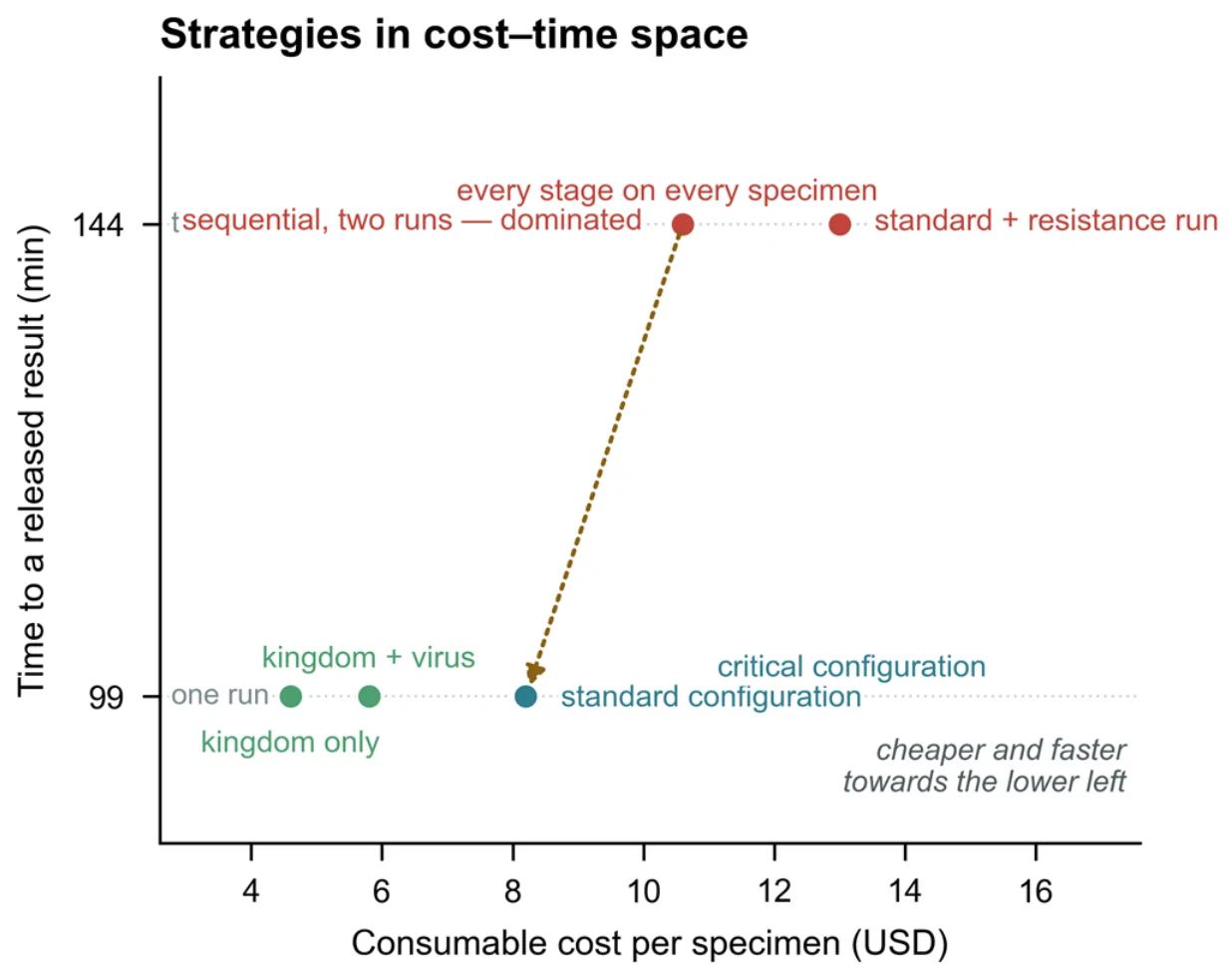
Strategies in cost–time space; cheaper and faster lie towards the lower left. Running the kingdom and species positions as two sequential runs is dominated by the standard configuration, which carries them together (dotted arrow). Colour marks what the strategy costs in runs: green a single run answering the kingdom question only, teal a single run that also carries organism positions, red a path that requires a second run.

**Table 1:** Previous reviews of artificial intelligence applied to antimicrobial resistance, and how this review differs. *Corpus appraised* is the body of work each review examined, not the number of records it retrieved. The right-hand column states what each design leaves unanswered rather than judging its quality: every one of these reviews answers the question it set. The last row applies the same columns to this current review.

Review	Scope	Corpus Appraised	Appraisal Instrument	Principal Finding	Not Addressed by The Design
Tang et al., 2022 [16]	Machine learning for antimicrobial resistance, any setting	25 studies	None reported	Pooled area under the curve 0.82 (0.78–0.85); development retrospective, validation scarce	Risk of bias; reporting completeness
Liu et al., 2024 [17]	Colonisation or infection with multidrug-resistant organisms, adults	162 models in 108 studies	PROBAST	High risk of bias, concentrated in the analysis domain; fewer than 25% externally validated, 7 of those independently	A single application niche; the artificial-intelligence extensions
Li et al., 2026 [18]	Multidrug-resistant infection in adult intensive care	100 models in 62 studies	PROBAST	High risk of bias in 87.1%; external validation in 17 studies	A single application niche; model availability; subgroup performance
Pu et al., 2025 [19]	Multidrug-resistant infection in comprehensive intensive care	37 models in 27 studies	PROBAST	Pooled area under the curve 0.867; calibration and external validation mostly absent	A single application niche; reporting completeness
Weis et al., 2020 [20]	Machine learning on MALDI-TOF mass spectra	36 studies	Study-level quality rating	External evaluation in 4 of 36 studies (11.1%)	A single modality; predates both extensions
Pennisi et al., 2025 [21]	Artificial intelligence in antimicrobial stewardship	80 studies	QUADAS-AI	Pooled area under the curve 72.3% (70.4–74.1)	Prediction-model conduct and reporting
Howard et al., 2026 [22]	Artificial intelligence across infectious diseases, resistance included	Narrative; no appraisal	None	Maps opportunities against the 2024 UN General Assembly targets	Methodological quality of the underlying studies
This review	Four domains read together: genomic prediction, clinical risk models, therapy and dosing, rapid diagnostics	725 mapped at abstract level; 80 appraised at full text	PROBAST+AI and TRIPOD+AI	External evaluation 45%, calibration 23%, a usable model 24%, sample size justified 16%; 8.8% call a random split external validation	No pooled performance estimate; a single coder; PubMed only

**Table 2:** Model classes compared across the 725 included studies (abstract level; classes are not mutually exclusive, since a study comparing several algorithms counts once in each). The right-hand block gives the same items read from the full text of the 80 appraised studies. The data-type column gives the most frequent category among the abstracts in which a data type could be assigned at all, and the column beside it gives the share in which it could not—54% in the regression group, which is why that cell should be read as the shape of what is reported rather than as a description of the class. The reinforcement-learning row is given as counts rather than percentages because it contains a single study: it learns β-lactam cycling policies from empirically measured fitness landscapes rather than predicting a phenotype for an individual patient, which is why it reports none of the three items, why no data type could be assigned to it from its abstract, and why it sits apart from the other classes. Em dashes in the right-hand block mark classes that contributed fewer than three studies to the appraised core set, where a proportion would not be interpretable. The underlying counts are given in Supplementary Table S8 at abstract level and Supplementary Table S8b at full text.

Model Class	n	% of 725	Most Frequent Domain	Most Frequent Classifiable Data Type	Data Type Not Assignable	External Validation	Calibration	Code Available	Appraised n	External Validation (Full Text)	Calibration (Full Text)	Risk of Bias Low
Regularised/classical regression	226	31.2%	Clinical/EHR	laboratory and microbiology	54.0%	18.6%	40.7%	2.2%	25	48.0%	52.0%	28.0%
Tree ensemble (random forest, boosting)	218	30.1%	Clinical/EHR	laboratory and microbiology	26.1%	15.1%	11.5%	5.0%	31	48.4%	22.6%	25.8%
Deep learning/neural network	147	20.3%	Rapid diagnostics	spectra, images, signals	14.3%	7.5%	2.0%	6.1%	18	44.4%	0.0%	11.1%
Support vector machine	60	8.3%	Rapid diagnostics	spectra, images, signals	20.0%	16.7%	8.3%	5.0%	10	60.0%	20.0%	30.0%
Unsupervised/clustering	30	4.1%	Rapid diagnostics	spectra, images, signals	20.0%	10.0%	6.7%	0.0%	—	—	—	—
Large language model	11	1.5%	Therapy and dosing	electronic health records	45.5%	9.1%	0.0%	9.1%	—	—	—	—
Reinforcement learning	1	0.1%	Genomic	not assignable	100%	0/1	0/1	0/1	—	—	—	—

**Table 3:** Consumable cost and time to a released result by path (developers’ reagent cost, exclusive of labour, instrument and overhead; robot-assisted timings). Each row is priced as 1.00 USD of releaser plus 1.20 USD per occupied position, controls included; the fourth, sixth and seventh rows describe a specimen run twice, buying two control pairs. Arithmetic in File S6b, timings in File S6c. What the unclosed channel budget of Section 4.8 would do to these costs and timings is given at the end of this section.

Path	Positions Occupied	USD	Released at (min)
Kingdom only, testing stops	1 + 2 controls	4.60	99
Kingdom + respiratory virus position	2 + 2 controls	5.80	99
Kingdom and species co-loaded, one run	4 + 2 controls	8.20	99
Co-loaded, then targeted resistance run	6 + 4 controls	13.00	144
Critical: kingdom, strain and resistance in one run	6 + 2 controls	10.60	99
Kingdom first, species in a second run	4 + 4 controls	10.60	144
Every stage on every specimen	7 + 4 controls	14.20	144

## Data Availability

No new data were created or analyzed in this study.

## Supplementary Materials

The following supporting information can be downloaded at https://www.mdpi.com/article/10.3390/microorganisms14102148/s1: It is grouped below by kind, and each entry names the file it was uploaded as, so that an item cited in the text can be matched to a file without opening it. Everything a claim in this paper rests on is here: the search as it was run, every screening decision, the appraisal of all 80 studies, the parameters and models behind Section 4, and two demonstrations that can be conducted rather than read. Search, screening and the evidence map. File S1 (S1_search_strategy.md, with the flow counts in S1b_prisma_flow.md): search strategy, the four full query strings as run, hit counts, retrieval dates, and the search log as written at search time—one record per query, carrying the run identifier, the timestamp, the number of records the query matched and the number retrieved—together with the PRISMA 2020 record counts behind Figure 1. Table S2 (S2_fulltext_exclusions.csv): the studies excluded at full text, with reasons. File S3 (S3_screening_decisions.csv): every screening decision with its reason, at record level. File S20 (S20_proportions_ci.py, with the temporal model in S20b_trend_model.py): the two analysis scripts, exactly as run. The first writes Table S14, the second Table S15; both read appraisal/appraisal_master.csv and search/evidence_map.csv from the reproduction package and depend only on pandas, NumPy and SciPy. File S17 (S17_prisma_2020_checklist.md): the PRISMA 2020 checklist, all 27 items, with the location of each in the manuscript or the supplement. Six shortfalls are declared. Four are answered *not done*—no protocol, no registration, no reporting-bias assessment and no certainty-of-evidence grading—and the remaining two, neither screening nor appraisal duplicated by a second human reviewer, are recorded against items 8, 9 and 11 as declared departures, because those items ask whether the process was described rather than who performed it. Table S4 (S4_evidence_map_725.csv): the evidence map across the 725 studies that carry the abstract-level analysis. Appraisal and analysis tables. Table S5 (S5_appraisal_80.csv): per-study PROBAST+AI and TRIPOD+AI appraisal records for the 80 core-set studies, including the domain judgments and their written rationales. Table S8 (S8_model_class_comparison.csv, with the appraised subset in S8b_model_class_appraised.csv): the model-class comparison underlying Table 2, at abstract level across all 725 studies and at full-text level across the 80 appraised studies. Table S14 (S14_proportions_ci.csv): every proportion reported in Section 3 with its Wilson score 95% confidence interval and the numerator and denominator it came from. Table S15 (S15_trend_model.csv): the four fits of the temporal model of Section 3.1—primary and sensitivity specifications at abstract level, and the same pair at full text—each with its odds ratio per year, profile likelihood interval and likelihood-ratio *p*-value. Table S16 (S16_qc_recoding.csv): the internal quality-control re-coding described in Section 5.5. Sixteen of the eighty appraised studies, drawn at random, were re-coded from full text without sight of the original records; the table gives both codings side by side, item by item, so that the agreement figures quoted in the limitations can be checked and the disagreements inspected. Table S16b (S16b_verification_pass.csv): the verification pass of Section 2.6, in full. All eighty studies were re-coded from full text on the four outcome-critical items—validation design, the accuracy of the authors’ own description of it, calibration, and subgroup performance—and every one of the 320 comparisons is given here with the quotation and the reason on each side, whether the two codings agreed or not, together with the written adjudication of each disagreement and a flag marking the studies whose original code the coder had seen before. File S22 (S22_analytical_validation_plan.md): the analytical validation plan for the platform of Section 4—extraction recovery, limit of detection per specimen type, pathogen and target, inhibition per position, inclusivity, exclusivity and cross-reactivity, multiplex interference target by target, polymicrobial competition, precision across operator, instrument, site, day and lot, stability and transport, carry-over, interfering substances and robustness—each with the acceptance criterion fixed in advance, and with the reverse-transcription step the respiratory RNA workflow requires and the manuscript had not specified. No study in it has been performed; it states what would have to be, and in what order. File S21 (S21_model_availability.md): model availability, TRIPOD+AI item 18e, coded from the full text of all eighty studies after external audit showed the rule-based coding failed in both directions. The file states the rule fixed before coding—an artefact from which a reader can obtain a prediction without refitting anything—gives the artefact class and the deciding quotation for every study, and, for every study coded negative that nonetheless carries a candidate signal, the reason the signal does not meet the rule. File S23 (S23_validation_basis.md): which clause of the Section 2.6 validation rule decided each of the eighty calls—place, time, source, a split of one assembled dataset, cluster resampling, other resampling, or no held-out evaluation at all—so that the rule can be checked in both directions rather than re-derived by each reader. It also records the two calls an external audit challenged and why they stand. File S24 (S24_open_access_basis.md): the licence or open-access statement quoted from each of the eighty full texts, with the rule that decided the call. The column this replaced was maintained by hand and recorded six of the eighty as closed; all six carry an explicit Creative Commons licence, which is why the basis is now published per study rather than asserted. Table S12 (S12_reference_audit_7d.csv): the seven-dimension citation audit of every reference—DOI, first author, full author list, journal, volume and issue, pages or article number, and PMID—each checked against a fresh PubMed record and against the Crossref record, with the database-formatting differences that are not defects recorded separately. Table S12b (S12b_reference_audit_6d_R1.csv): the same audit re-run over the printed reference list of this revision, covering all 145 entries, with the deciding authority recorded for each. Platform specification and models. Table S9b (S9b_configuration_library.csv): the manufactured configuration library of Section 4.4—every configuration with its specimen and control positions, its contents, whether it carries the reserved core block, whether it exists only as a second run, its consumable cost and its intended use. Generated from the same configuration object that prices the paths in Table 3, so the library and the prices cannot disagree. File S6 (S6_time_budget.csv, with the consumable model in S6b_cost_model.csv, the per-path timings in S6c_path_timings.csv and the strategy comparison in S6d_triage_strategies.csv): the step-by-step time budget of Section 4.5 with each step marked as on or off the critical path, the consumable cost model behind Table 3 under both control-accounting conventions, the total time for every combination of run outcomes, and the four panel-selection strategies of Section 4.6 written out branch by branch—each branch with its probability, the path it takes, its cost and its time, and the population mean they produce—so that the 6.04, 8.20 and 7.00 USD means and the sensitivity of the first to M1’s accuracy can be recomputed rather than taken on trust. All four are generated from platform/platform_config.json, which is the single place the platform’s unit costs, timings and configuration library are recorded. Table S9 (S9_design_parameters.csv): every primary design parameter behind Section 4.5 and Table 3 with its provenance—measured in the authors’ own laboratory, instrument or vendor specification, planning assumption, or design decision. Table S10 (S10_model_specification.csv): the per-module specification for the seven decision points—model class, inputs, output, calibration requirement, validation design under TRIPOD+AI, primary failure mode, safeguard, and the decision that stays human. Assay design document. File S7 (S7_kingdom_targets_and_virulence.md): the six channel assignments of the group-determination tube, kingdom-specific primer targets with their published validation and the constraints that determine whether each channel can be believed, the route-specific escalation menus, secondary discriminating targets and the kingdom-specific virulence-factor menus supporting Section 4.3. File S18 (S18_model_flowline.png): where the models act along the working sequence, from sampling to prevention and follow-up, with the seven model decisions above the spine and the decisions that stay with a person—releasing a result, narrowing a panel, writing a prescription, an administering microbiota restoration—below it. Supporting Section 4.7. File S19 (S19_failure_mapping.md): each failure measured in the appraisal, its frequency across the eighty studies, and the design response Section 4 makes to it. The frequencies are computed from the appraisal records rather than transcribed. Interactive demonstrations. Both are single self-contained HTML files that open in any current browser, require no installation and make no network request. Neither carries analytical or clinical validation, and neither is a medical device. File S11 (S11_decision_support_demo.html): an illustrative implementation of the four-step sequence—presenting syndrome and laboratory values, panel selection, the qPCR result, targeted therapy with dose, interval and duration, and *Clostridioides difficile* prevention—in which every agent exclusion is traceable to the rule that produced it, and consumable cost and turnaround are computed from the same unit prices and stage durations as Table 3 and File S6 rather than quoted. File S13 (S13_live_sample_run_clock.html): the live-sample run clock, an animation of the time budget that plays the manufactured strip configurations and the escalation path against a 45-cycle run, reaching each model decision at the minute it is reached and showing the reflex trigger at cycle 22 and the completed run side by side, so that what the crossing does and does not authorise can be watched rather than read. Figures. Figure 1, Figure 2, Figure 3, Figure 4, Figure 5, Figure 6 and Figure 7 are supplied at 600 dpi as LZW-compressed TIFFs and as editable SVGs, in which every label remains live text rather than an outline.
