# Supplementary material for: Validation, Calibration and Transparency, More than Accuracy: An Appraisal of Artificial Intelligence Against Antimicrobial Resistance and a Sustainable, Testable Diagnostic Architecture
Source: Microorganisms. 2026 Sep 23;14(10):2148. doi: 10.3390/microorganisms14102148 (PMC13707508; doi:10.3390/microorganisms14102148)
Supplement: Supplementary file 1 [file microorganisms-14-02148-s001.zip › microorganisms-4564098-supplementary/S11_decision_support_demo.html]

Panel to Prescription


Live-sample platform · decision-support demonstration

# Symptoms and laboratory values to a targeted prescription

Four steps in the order they happen at the bedside. The models choose the panel before the
specimen is loaded, the run answers, therapy becomes targeted rather than empirical, and
*Clostridioides difficile* risk is addressed at the point where the antibiotic decision
is actually made.

**Demonstration of decision logic only. Not a medical device and not for patient care.**
The rules encoded here are a simplified, illustrative subset of adult empirical guidance, with
no analytical or clinical validation. A genotype shifts a probability; it does not replace
phenotypic susceptibility testing. Every prescription is a clinician's decision.

Worked cases

1

## Symptoms and laboratory values

before the specimen is loaded · M1, M2

Presenting syndrome

Age (y)

Weight (kg)

SexMaleFemale

Temp (°C)

Systolic BP

Resp. rate

CRP (mg/L)

PCT (µg/L)

Lactate (mmol/L)

WBC (×10⁹/L)

Creatinine (µmol/L)

β-lactam allergy
None
Non-severe rash
Anaphylaxis

Known MRSA colonisation or previous MRSA
 Previous ESBL or recent broad-spectrum β-lactam
 Structural lung disease, recent ICU stay or indwelling device
 Neutropenia or significant immunosuppression
 Prior *C. difficile* infection, or ≥2 antibiotic courses in 3 months

2

## qPCR run

enter C_t_, blank = no amplification · M0

3

## Targeted therapy, dose and duration

no longer empirical · M3, M4

4

## *Clostridioides difficile* prevention

decided with the antibiotic, not after it · M5

**Why the order matters.** Steps 1 and 3 both produce an antibiotic recommendation, and
they are not the same recommendation. Step 1 is a hedge against not knowing the organism, and
in sepsis it must not wait — guidance requires therapy within the hour. Step 3 is a choice
made with the organism and its determinants in hand. The distance between the two is the
quantity this platform exists to shorten: not time to the first dose, but time to the first
*appropriate* one.

**Why every exclusion is shown.** The trace under each result is the point of the design.
A recommendation whose reasons cannot be read is one a microbiologist cannot check at three in
the morning, and Section 3 of the accompanying review found that most published models in this
field cannot be checked at all.
