# Supplementary material for: Validation, Calibration and Transparency, More than Accuracy: An Appraisal of Artificial Intelligence Against Antimicrobial Resistance and a Sustainable, Testable Diagnostic Architecture
Source: Microorganisms. 2026 Sep 23;14(10):2148. doi: 10.3390/microorganisms14102148 (PMC13707508; doi:10.3390/microorganisms14102148)
Supplement: Supplementary file 1 [file microorganisms-14-02148-s001.zip › microorganisms-4564098-supplementary/S13_live_sample_run_clock.html]

Live-Sample Run Clock


Live-sample platform · one specimen, end to end

# From specimen to released recommendation

The clock runs in platform minutes on the measured robot-assisted budget:
24 minutes pre-analytical, 15 minutes of reaction preparation, amplification, and 15 minutes
post-analytical. Every run goes to cycle 45. A target crossing threshold at cycle 22 of 45
triggers the reflex chain halfway through the run and authorises a second configuration to be
charged, but it releases nothing — at cycle 22 the no-template control is clean only so
far and the internal amplification control has not yet crossed. Every channel, crossed or flat,
becomes reportable only when the run finishes with all controls valid.

Pause

Triage
Standard
Standard + escalation
Critical

Robot-assisted
Manual

1×
2.5×
6×

Elapsed0 min

Released at—

Consumable cost—

Positions occupied—

One-hour sepsis rule—

Stage clock

Eight-position lyophilised configuration — standard

Amplification, six optical channels


Cycle 0 of 45

Decisions, as they are released

**What the animation is driven by.** Stage durations come from the project's time budget
(platform/time\_budget.py) and the path totals from the staged run model
(platform/cost\_model.py, from
platform/platform\_config.json): 99 minutes for any single
configuration, since every run is carried to completion before anything is reported, and 144
when a reflex second run follows. Consumable figures are the
developers' own reagent costs — releaser 1.00 USD per specimen, 1.20 USD per lyophilised
position, two control positions per manufactured configuration — not a price to any
payer.

**The one-hour rule is not a finish line the platform has to beat.** In sepsis, empirical
therapy starts within the hour regardless; the platform is not permitted to gate that first dose.
What it shortens is the time to the first *appropriate* dose.

**Where the selection minute sits.** The one minute in which the models choose which
manufactured configuration comes off the shelf runs concurrently with plate closure, so it is
drawn inside reaction preparation rather than added to it. The models do not choose which tubes
of a strip are wetted: the reagent is committed at manufacture.

**A crossing at cycle 22 is a reflex trigger, not a release.** At cycle 22 the positive
control has crossed, at cycle 19, but the two controls that would license a report have not
finished their work. The no-template control is clean only *so far*: contamination
frequently amplifies late, and a detectable no-template signal at any cycle invalidates the
run, so a clean trace at cycle 22 is a provisional state and not a result. The internal
amplification control is designed to cross near cycle 28, so it has not amplified either, and
every channel that is still flat is uninterpretable. Nothing is reportable until the run has
finished with all controls valid — a negative never was, and on this policy a positive
is not either.

**What the crossing does buy** is the next step: the advisory chain starts and a second
manufactured configuration can be authorised and charged inside the 23 cycles the first run
still has to go, so a reflex run adds 45 minutes rather than 50. The first run is *not*
stopped. Stopping it would give up the channels that had not yet crossed, and in a
polymicrobial or undifferentiated specimen those channels may carry a second pathogen —
which is a diagnostic loss no gain in minutes justifies.
